# Structural Integrity of Nucleolin Is Required to Suppress TDP-43-Mediated Cytotoxicity in Yeast and Human Cell Models

**DOI:** 10.3390/ijms242417466

**Published:** 2023-12-14

**Authors:** Caterina Peggion, Maria Lina Massimino, Daniel Pereira, Sara Granuzzo, Francesca Righetto, Raissa Bortolotto, Jessica Agostini, Geppo Sartori, Alessandro Bertoli, Raffaele Lopreiato

**Affiliations:** 1 Department of Biology, University of Padova, 35131 Padova, Italy; 2 Neuroscience Institute, Consiglio Nazionale Delle Ricerche, 35131 Padova, Italy; 3 Department of Biomedical Sciences, University of Padova, 35131 Padova, Italy; 4 Department of Bioengineering, iBB—Institute for Bioengineering and Biosciences, Instituto Superior Técnico, University of Lisbon, 1049-001 Lisbon, Portugal; 5 Padova Neuroscience Center, University of Padova, 35131 Padova, Italy

**Keywords:** neurodegenerative disorders, TDP-43 proteinopathies, nucleolin, nucleocytoplasmic trafficking

## Abstract

The Transactivating response (TAR) element DNA-binding of 43 kDa (TDP-43) is mainly implicated in the regulation of gene expression, playing multiple roles in RNA metabolism. Pathologically, it is implicated in amyotrophic lateral sclerosis and in a class of neurodegenerative diseases broadly going under the name of frontotemporal lobar degeneration (FTLD). A common hallmark of most forms of such diseases is the presence of TDP-43 insoluble inclusions in the cell cytosol. The molecular mechanisms of TDP-43-related cell toxicity are still unclear, and the contribution to cell damage from either loss of normal TDP-43 function or acquired toxic properties of protein aggregates is yet to be established. Here, we investigate the effects on cell viability of FTLD-related TDP-43 mutations in both yeast and mammalian cell models. Moreover, we focus on nucleolin (*NCL*) gene, recently identified as a genetic suppressor of TDP-43 toxicity, through a thorough structure/function characterization aimed at understanding the role of NCL domains in rescuing TDP-43-induced cytotoxicity. Using functional and biochemical assays, our data demonstrate that the N-terminus of NCL is necessary, but not sufficient, to exert its antagonizing effects on TDP-43, and further support the relevance of the DNA/RNA binding central region of the protein. Concurrently, data suggest the importance of the NCL nuclear localization for TDP-43 trafficking, possibly related to both TDP-43 physiology and toxicity.

## 1. Introduction

The Transactivating response (TAR) element DNA-binding of 43 kDa (TDP-43) protein belongs to the large family of heterogeneous nuclear ribonucleoproteins (hnRNP) [1], ubiquitously expressed in tissues and mainly localized within the cell nucleus, although it can shuttle to the cytoplasm to form cytoplasmic RNP granules [2]. Starting from its initial discovery as a protein that binds to TAR DNA, a specific region in the HIV genome [3], further research revealed that TDP-43 regulates multiple cellular processes, such as gene transcription, mRNA splicing, stability, translation, trafficking, and miRNA synthesis [4,5,6]. In normal conditions, TDP-43 acts also on its own RNA to regulate its abundance via a negative feedback loop, which causes mRNA degradation [7].

In humans, TDP-43 protein, encoded by *TARDBP* gene, is composed of 414 amino acids (aa) (Supplementary Figure S1), consisting of a globular N-terminal domain (aa 1–81) that participates in self-oligomerization [8], a nuclear localization signal (NLS, aa 82–106), two tandem RNA-recognition motifs (RRMs) (aa 106–292) required to bind nuclear transcripts, a nuclear export signal (NES, aa 239–274), and a C-terminal glycine-rich low complexity domain (CTD, aa 275–414) associated with prion-like properties [9,10] and responsible for alternative splicing function, protein–protein interaction [5,11,12,13,14], and for the recruitment of the protein in stress granules. Like other prion-like domains, the CTD of TDP-43 is intrinsically disordered and extremely aggregation-prone, playing a crucial role in TDP-43 phase transition, such as its dispersion into liquid droplets and its accumulation into irreversible cytoplasmic aggregates [9,15,16,17,18].

Because of its cellular relevance, TDP-43 perturbations in either phase transition properties, autoregulation mechanism, and/or nucleocytoplasmic trafficking are causative of both loss of its normal functionality and the toxic gain of function of TDP-43 aggregates, finally resulting in abnormal RNA/DNA metabolism.

In this context, the nuclear clearance of TDP-43 and the accumulation of cytoplasmic TDP-43 aggregates represent the core pathobiology of TDP-43 proteinopathies, including almost all cases (97%) of amyotrophic lateral sclerosis (ALS) [19,20] and a considerable number of cases of frontotemporal lobar degeneration (FTLD) [21], comprising one of its clinical subgroups known as frontotemporal dementia (FTD) [22], and of Alzheimer’s disease [23]. Importantly, the identification of several missense mutations in the *TARDBP* sequence in familial cases of both ALS and FTLD [24,25], most of which (~90%) occurred in the region coding for CTD and promoted the intrinsic aggregation of the protein, provided an exciting link between the pathophysiology of apparently sporadic and familial diseases and contributed to unveiling pathological molecular pathways [24].

Our knowledge of TDP-43′s functions, interactions with other cellular components, and the cellular effects of TDP-43 dysfunction have all been greatly improved by research on the protein in yeast cell models [26]. In particular, the ectopic expression of human wild-type (WT) or disease-associated mutant forms of *TARDBP* gene is toxic and forms TDP-43 cytoplasmic aggregates in yeast cells, providing the opportunity to shed light on how the protein may affect cell viability and RNA metabolism [26,27,28]. Moreover, yeast models were successfully applied to perform screening for genetic modifiers or potential therapeutic targets that could influence TDP-43 aggregation and/or toxicity [29,30,31].

Recently, we established novel models of TDP-43 toxicity in yeast [32], based on the finely tuned expression of *TARDBP* gene, either WT or bearing specific ALS-related missense mutations (i.e., Q331K and M337V). Interestingly, using such models, we demonstrated that the expression of the human nucleolin gene (*NCL*) counteracts TDP-43-dependent toxicity, which was further confirmed in mammalian HEK293T cells. Although the molecular mechanism(s) were yet undefined, data indicated the ability of NCL to restore the physiological localization and the functionality of TDP-43 [32].

NCL is an abundant and ubiquitously expressed shuttling protein that is located principally in the nucleolus but also moves to the nucleoplasm, cytoplasm, and plasma membrane, where it has been implicated in multiple cellular functions [33,34,35,36,37]. Indeed, NCL is defined as a multifunctional protein that participates in different steps of RNA/DNA metabolism (such as the regulation of RNA polymerase I transcription, folding and maturation of the pre-ribosomal RNA (pre-rRNA), and ribosome assembly [33,34,35,36,37]). Furthermore, NCL has been designated as a histone chaperone and chromatin remodeler, also participating in the regulation of translation and mRNA stability, as well as the import and/or export of several nucleolar components or proteins. Moreover, NCL contributes to cell migration, adhesion, and virus infection by moving to the cell membrane [37].

The structure of human NCL consists of 710 aa and can be divided into three main regions, displaying different signatures and functional roles: the intrinsically disordered N-terminal portion (aa 1–300), followed by a central region (aa 300–650) carrying four RNA-Recognition Motif (RRM) domains, and the intrinsically disordered C-terminal portion (aa 650–710) rich in glycine/alanine residues (GAR) [35]. The N-terminal domain is the site of numerous post-translational modifications and contains four highly acidic stretches necessary for the interaction with basic proteins such as histones and eight tandem repeats ([ST]-P-x-K[KA]) which can be recognized by proline-directed kinases and are involved in NCL localization [37]. The four RRM domains are highly conserved and essential for protein function [38], allowing the specific interaction of NCL with mRNA and rRNA, further promoting its nucleolar accumulation. The C-terminal GAR domain facilitates the interaction with RNAs and ribosomal proteins and is also required for the nucleolar localization of NCL [39,40,41]. Importantly, NCL translocation to the nucleus occurs via the specific signal sequence (NLS) positioned at the end of the N-terminal region (aa 279–298) [40]. However, NCL movements to other cell compartments seem to be also regulated by the GAR domain [42].

In this work, to deeply characterize the safeguard properties of NCL against TDP-43 toxicity, we use yeast and mammalian cell models to investigate the ability of NCL to counteract the toxicity induced by the overexpression of some TDP-43 variants specifically associated with FTLD. Additionally, we examine the effects of deleting different portions of NCL in order to understand their role in rescuing the TDP-43-associated lethal phenotype. Our data demonstrate that the N-terminal and central regions of NCL are essential for the protein to exert its antagonizing effects on TDP-43, indicating that almost the entire NCL is crucial to play such task. Moreover, data also suggest that NCL participates in TDP-43 nuclear import thanks to the maintenance of its NLS sequence.

## 2. Results

### 2.1. FTLD-Related TDP-43 Mutants Are Toxic to Yeast Cells, but Cured by NCL Expression

In this study, we extended the use of yeast models to investigate the effect of known mutations related to FTLD-TDP-43 proteinopathies, such as the G295S, M359V, and A90V replacements [43,44,45]. Therefore, these point mutations were introduced in the *TARDBP* coding sequence already cloned in the galactose-inducible plasmid pRS246GalTDP43-GFP. Recombinant plasmids were then used to transform the yeast CEN.PK strain and to perform the functional assays.

**Figure 1:**
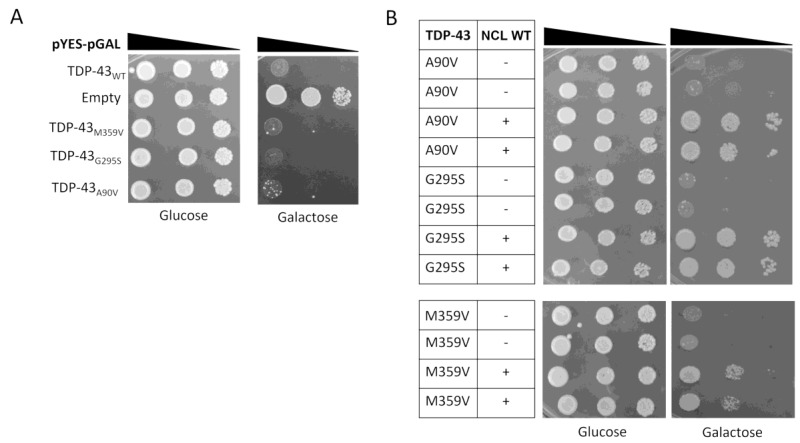
Cytotoxicity of FTLD-related TDP-43 mutants in yeast cells can be rescued by the expression of the *NCL* gene. (**A**) Growth assay of *S. cerevisiae* CEN.PK strain transformed with the plasmids encoding for either wild-type (WT) or mutant (M359V, G295S, A90V) TDP-43. Cells transformed with the empty vector were considered as control. (**B**) CEN.PK cells carrying the plasmids encoding the FTLD-TDP-43 mutants were co-transformed with either multi-copy plasmid expressing the wild-type (WT) isoform of *NCL* (+), or the empty vector as negative controls (-). Yeast cells (OD_600_ = 1) of each strain were serially diluted (10-fold) and spotted onto either repressing condition as control (glucose), or in inducing medium plates (galactose) and incubated at 30 °C for several days (3 for glucose and 5 for galactose). Data are representative of three independent experiments with similar results.

As shown in Figure 1A, the overexpression of either WT or FTLD-related TDP-43 mutants in galactose medium (right panel) caused the lethality of the yeast cells, whereas no effects were observed by growing yeast strains in non-inducing conditions, thus confirming that comparable amounts of living cells were present in all samples (i.e., glucose medium, left panel). Such data supported the notion that the yeast model may be useful for studying TDP-43 dysfunctions (i.e., TDP-43 proteinopathies) other than those related to ALS.

Interestingly, the overexpression of human WT *NCL* protected the yeast cells against the toxicity induced by the expression of *TARDBP* mutant forms (Figure 1B), as already observed for both WT and ALS-associated TDP-43 mutants [32].

Data thus indicated that NCL is able to trigger beneficial effects toward both ALS- and FTLD-related TDP-43 cytotoxicity, pointing to the determination of the molecular mechanism(s) at the basis of such ability.

### 2.2. Analysis of NCL Mutants in S. cerevisiae Yeast Cells

Identifying NCL regions essential to its safeguarding role against TDP-43-induced cytotoxicity could elucidate the mechanisms of such an effect. To this purpose, we generated different truncated mutants of NCL starting from a galactose-inducible yeast-expressing plasmid encoding WT NCL (full-length, 710 aa) fused to the red fluorescent mKate2 protein (Table 1).

We checked the expression of NCL mutant isoforms in CEN.PK yeast strain transformed with the different constructs and grown in galactose for 24 h using Western blot (WB) analysis. As shown in Supplementary Figure S2, both WT and mutant NCL proteins displayed electrophoretic mobility profiles with higher molecular mass than the theoretical ones (Table 1), also considering the presence of the mKate2 fusion protein. Such upward shifts were likely caused by post-translational modifications that are known to occur in NCL post-translational processing (e.g., glycosylation). As previously reported, additional bands at lower molecular weights probably reflected proteolytic degradation processes [32,37,46,47].

**Figure 2:**
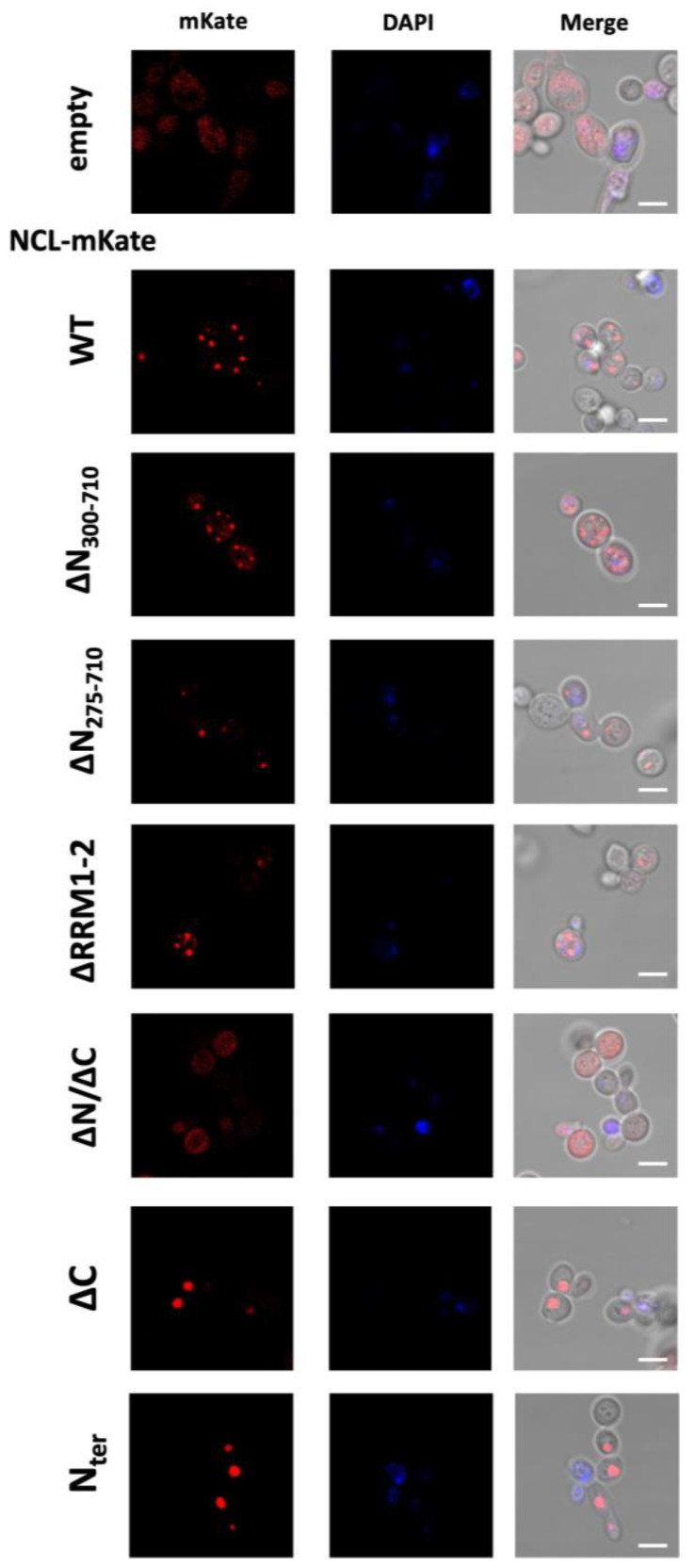
Localization of NCL mutant isoforms in yeast cells. CEN.PK cells were transformed with multi-copy plasmids expressing the mKate2 red fluorescent protein alone (empty) or fused to either NCL full-length protein (WT) or the indicated mutant isoforms. After 24 h of incubation in inducing (galactose) medium, yeast cells were fixed and analyzed with confocal microscopy to observe the distribution of NCL-mKate2 fusion proteins (**left**), the DAPI staining (**middle**), and the merged signals with the cellular morphology (using DIC, *differential interference contrast*) (**right**). Images represent yeast cells enlargements taken from fields reported in Supplementary Figure S3. Three biological replicates for each strain were analyzed. Scale bar, 5 μm.

Since data confirmed the expression of the different NCL protein isoforms in yeast cells, we further investigated the effect of the mutations on NCL cellular localization using confocal microscopy, thanks to the presence of the mKate2 red fluorescent protein, and representative images are shown in Figure 2.

As already observed [32], full-length NCL (WT) distribution in yeast was characterized by a punctated localization, forming several fluorescence foci arranged in the periphery of the cell. Although with lower signal intensity, similar fluorescence patterns were observed for the NCL mutants lacking either the N-terminal region (∆N_300–710_, ∆N_275–710_) or the central DNA/RNA-binding domains RRM1–2 (NCL ∆RRM1–2). Conversely, the mKate2-fused mutant isoform without the C-terminus (NCL ∆C), as well as the NCL N-terminus alone (NCL N_ter_), were massively accumulated in a single (large) fluorescent structure.

Such findings indicated that the NCL structural modifications perturbed the protein’s distribution in yeast cells compared to full-length NCL, although in different manners depending on the deleted regions.

**Table 1:** Schematic representation of the NCL mutant isoforms with the amino acid coordinates and the expected dimensions (MW) for the different NCL polypeptides. Reported MW considered only the dimension of NCL protein. To obtain the size of the NCL-mKate2 fusion proteins, around 27 kDa must be added to the indicated values (for a schematic representation of the mutations, see Figure 3).

	NCL	Coordinates (aa)	MW (Theoretical)
**1**	WT (full-length)	1–710	76.5
**2**	ΔN_300–710_	300–710	44.7
**3**	ΔN_275–710_	275–710	47.5
**4**	N_ter_	1–300	32.2
**5**	ΔC	1–650	70.7
**6**	ΔN/ΔC	300–650	38.9
**7**	ΔRRM1–2	1–300//471–710	57.8

### 2.3. Effects of NCL Mutations on Its Ability to Suppress TDP-43-Dependent Toxicity in Yeast Cells

The *S. cerevisiae* strain CEN.PK-TDP 2C, carrying two chromosomal copies of the *TARDBP* gene under the control of the *GAL1* promoter, was used to test the capability of NCL mutants to recover TDP-43-induced toxicity. This strain has been already characterized [32], demonstrating the toxic effect of TDP-43 expression and further unveiling the ability of WT NCL to revert TDP-43-induced cell death. Hence, CEN.PK-TDP 2C cells were transformed with the plasmids expressing the NCL deletion mutants or with either the *NCL* WT (full-length) isoform or the empty vector (encoding mKate2 only) as positive or negative controls, respectively, and functional (spot) assays were performed to assess yeast cell viability. Notably, the expression of *NCL* mutants was not perturbed in yeast cells co-expressing *TARDBP* (Supplementary Figure S4).

**Figure 3:**
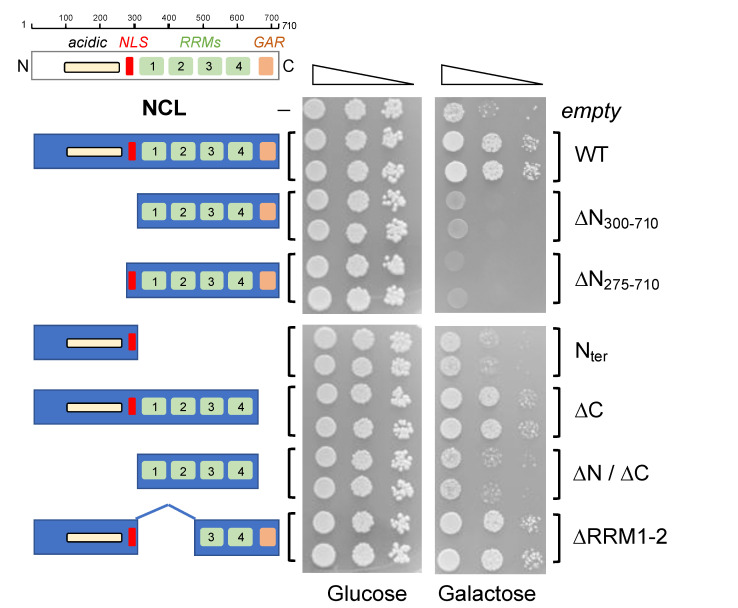
Effects of the NCL mutations on its ability to alleviate the TDP-43-dependent cytotoxicity in yeast cells. Growth assay of *S. cerevisiae* CEN.PK TDP-2C strain (carrying two copies of *TARDBP* transgene) transformed with the plasmids encoding for either NCL wild-type (WT) or the indicated mutant isoforms. Cells transformed with the empty vector were considered as controls. Yeast cells (OD_600_ = 1) of each strain were serially diluted (10-fold) and spotted onto either repressing condition as control (glucose) or in inducing medium plates (galactose) and incubated at 30 °C for several days (3 for glucose, 5 for galactose plates). Data are representative of three independent experiments with similar results.

As shown in Figure 3, no differences in cell viability were observed between the strains in permissive conditions (glucose panel), where neither TDP-43 nor NCL were produced, thus indicating that similar number of living cells were present in each sample. Instead, under conditions that induced the expression of both *TARDBP* and *NCL* (galactose panel), the mutations introduced in the NCL sequence caused rather different effects on the protein’s ability to counteract the toxicity caused by TDP-43.

Indeed, the loss of the N-terminal region of the protein (NCL ∆N_275–710_ and ∆N_300–710_ mutants), regardless of the presence of the native nuclear localization signal (NLS), caused the loss of the capability to reduce cell death, strongly indicating that the NCL N-terminal region was necessary to suppress TDP-43 toxicity. This notion was further supported by the similar behavior of the NCL mutant consisting of the middle portion of the protein (i.e., the RRM domains, NCL ∆N/∆C). However, the expression of only the N-terminal region (*NCL* N_ter_) was not able to counteract TDP-43-induced cell death, thus indicating that despite being necessary, the N-terminus of the protein was not sufficient for the safeguard activity of NCL. On the contrary, the loss of the disordered C-terminal tail (NCL ∆C) did not perturb the curative effects of NCL, with respect to the WT isoform, therefore excluding its implication in the NCL ability to suppress TDP-43-dependent cytotoxicity. Interestingly, a similar behavior was observed following the partial deletion of the NCL middle region (i.e., two out of four RRM domains, NCL ∆RRM1–2), which did not significantly modify the protective function of the protein.

Very similar results were further observed in CEN.PK cells expressing both TDP-43 and the different isoforms of NCL proteins by multicopy plasmids (Supplementary Figure S5), collectively suggesting that the N-terminal domain of NCL was required but insufficient to reduce the toxicity of TDP-43, even when the latter protein was expressed at very high levels.

Taken together, data suggested that the molecular mechanisms at the basis of NCL’s ability to cure TDP-43-dependent toxicity strictly implicated the N-terminal region, but further pointed (at least partially) to NCL’s ability to interact with nucleic acids via the central portion carrying the RRM domains.

We also analyzed the cellular distribution of TDP-43-GFP and the different NCL-mKate2 mutants in yeast CEN.PK co-transformed cells with confocal microscopy. Although some overlapping between the green and the red fluorescent signals was observed with a variable frequency for the different NCL isoforms (Supplementary Figure S6), no clear correlation between TDP-43/NCL colocalization and the suppressive activity of NCL variants against TDP-43 toxicity emerged from these experiments.

### 2.4. Evaluation of Toxicity of FTLD-Related TDP-43 G295S Mutation in a Human Cell Model

**Figure 4:**
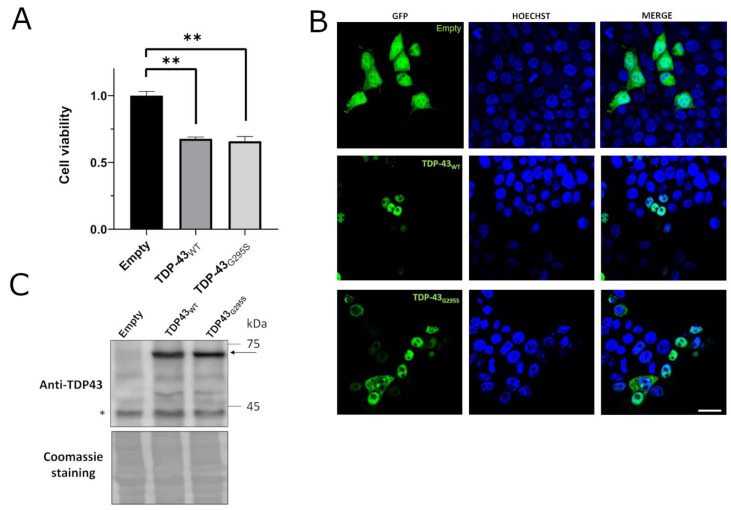
The production of TDP-43 G295S mutant-induced cell death in HEK293T cells. (**A**) MTS assay performed in HEK293T cells transiently transfected with plasmids encoding GFP-fused TDP-43 WT and G295S mutant (dark and light grey bars). As a control, MTS assay was performed on cells transfected with plasmid encoding GFP only (empty). Data were normalized to the mean value of the control sample. *n* = 7, ** *p* < 0.01, Kruskal–Wallis test followed by a Dunn’s post hoc test. (**B**) Representative confocal images of HEK293T cells transfected as in panel A. Scale bar 25 µm. (**C**) WB analysis of HEK293T cells transfected as in panel A using antibody against TDP-43. The arrow indicates the proper molecular weight of TDP-43-GFP chimeric proteins, while the asterisk represents the band corresponding to the endogenous protein. As control of loading, the Coomassie staining of the membrane is shown.

To verify whether the FTLD-related TDP-43 G295S mutation triggered cytotoxicity in mammalian cells, the human cell line HEK293T was transiently transfected with the plasmids encoding fluorescence-tagged WT or G295S mutant TDP-43-GFP, and the viability was compared with cells transfected with the empty vector. As shown in Figure 4A, the ectopic production of TDP-43 G295S caused the same significant reduction (approximately 40%) in cell viability as the WT protein (see also [32]), confirming, in human cells, the cytotoxic potential of high TDP-43 levels. Expression and localization of TDP-43 were verified via both immunofluorescence and WB (Figure 4B,C).

### 2.5. NCL Mutations Perturbed Its Ability to Alleviate TDP-43 Cytotoxicity in HEK293T Cells

As previously reported [32], full-length NCL was able to suppress the cytotoxicity caused by TDP-43 in HEK293T cells. Here, we investigated the protective role of NCL against the production of the TDP-43 G295S mutant and the impact of some deletion mutations on the role of NCL as a toxicity suppressor in mammalian cells.

Bearing in mind our observations in yeast cells, we focused on NCL mutants either depleted of the N-terminal region (∆N_300–710_, and ∆N_275–710_) or the NCL N-terminus only (NCL N_ter_), all of which completely lost curative abilities in the yeast model (see Figure 3).

To this purpose, the different isoforms of *NCL*-mKate2 (WT and mutants) were cloned into a pcDNA3 mammalian vector and the expression and intracellular localization of the different proteins were tested by transiently transfecting HEK293T cells and by performing WB and confocal imaging assays, respectively (Supplementary Figure S7). As expected, overexpressed WT NCL was mainly localized in the nucleolar compartment, while NCL truncated isoforms were characterized by different cellular localization: the ∆N_300–710_ mutant, lacking the NLS sequence, showed a predominantly extranuclear localization and both ∆N_275–710_ and N_ter_ mutants displayed promiscuous nucleolar/cytosolic and nucleolar/nuclear localization, respectively (Supplementary Figure S7, panel A).

**Figure 5:**
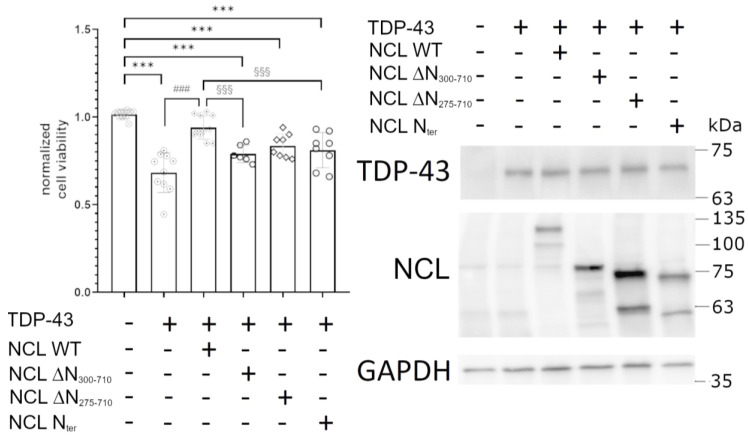
Cell viability of HEK293T cells transiently expressing vectors encoding for TDP-43-GFP G295S mutant and different isoforms of NCL-mKate2 (+). As a control, cells were transfected with GFP or mKate2-encoding plasmid. Data were normalized to the mean value of control samples (first bar), transfected with plasmids encoding mKate2 and GFP only. n is reported as symbols in the bars; *** *p* < 0.001, vs. control samples, ### *p* < 0.001 vs. cells transfected with plasmid encoding TDP-43-GFP and mKate2 (second bar), §§§ *p* < 0.001 vs. cells transfected with TDP-43-GFP and NCL WT, Kruskal–Wallis test followed by a Dunn’s post hoc test (**left panel**). In the (**right panel**) is shown a representative WB out of three biological replicates (i.e., different cell transfections) of HEK293T cells co-transfected as in the left panel using antibodies to GFP and mKate2 (recognizing the TDP-43-GFP and NCL-mKate2 chimera, respectively) and to GAPDH employed as loading control.

To compare the ability of WT and mutant NCL isoforms to suppress TDP-43 G295S-induced cell death in HEK293T cells, we assessed the viability of cells transiently co-transfected with both plasmids encoding the TDP-43 G295S mutant and each different NCL isoform. As shown in Figure 5 (left panel), cells overexpressing mutant TDP-43 were significantly less viable (approximately 40%) compared to control cells (co-transfected with empty plasmids, only encoding GFP and mKate2). The co-expression of full-length NCL significantly rescued cells from TDP-43 G295S toxicity, while all mutated proteins clearly lost suppressive abilities, thus indicating that the N-terminus of the NCL protein was required, but not sufficient, for full protection in human cells. The production of TDP-43 and all NCL isoforms by the plasmid vectors was confirmed by WB data (Figure 5, right panel). Importantly, the single expression of each *NCL* isoform did not affect cell viability, as reported in Supplementary Figure S8.

**Figure 6:**
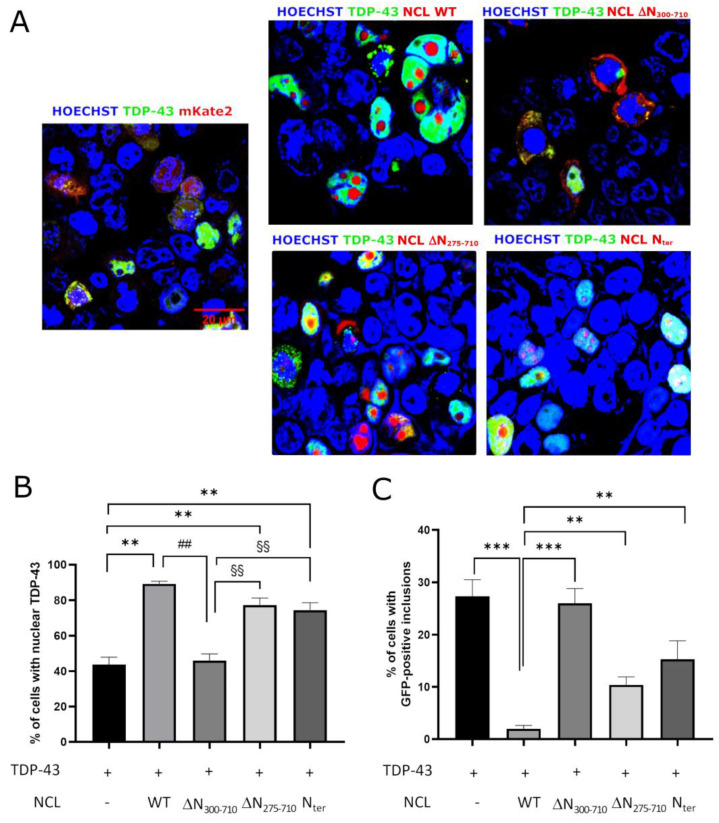
Effects of NCL mutations on TDP-43 cellular localization. (**A**) Confocal microscopy analysis of HEK293T cells co-transfected with plasmids coding for GFP-fused TDP-43 G295S mutant and either mKate2 alone (TDP-43 + mKate2, left panel) or the different NCL-mKate2 chimeras (TDP-43 + NCL WT, central, top panel; TDP-43 + NCL ΔN_300–710_, right, top panel; TDP-43 + NCL ΔN_275–710_, central, bottom panels; TDP-43 + NCL N_ter_, right, bottom panel) and counterstained with the nuclear marker Hoechst. The overlay of GFP/mKate2 and Hoechst signals is shown. Raw images (i.e., not electronically or digitally zoomed and not merged) are shown in Supplementary Figure S9. (**B**) Percentage of cells producing TDP-43-GFP G295S and mKate2 (–) or different isoforms of NCL-mKate2 with an exclusively nuclear GFP signal, with respect to the total of cells co-expressing both proteins. n = 3; **, ##, §§ *p* < 0.01, one-way ANOVA followed by Sidak’s multiple comparison test. (**C**) Percentage of cells co-expressing vectors encoding for TDP-43-GFP, G295S, and mKate2 (-) or different isoforms of NCL-mKate2 presenting discrete GFP aggregation-like foci, with respect to the total of cells co-expressing both proteins. n = 3; ** *p* < 0.01, *** *p* < 0.001, one-way ANOVA followed by Sidak’s multiple comparison test.

We further evaluated the cellular distribution of GFP-fused TDP-43 G295S protein (Figure 6, panel A), as well as the presence of TDP-43 inclusions, in HEK293T cells co-transfected with the TDP-43 G295S plasmid and with either the empty vector (encoding mKate2, used as control) or plasmids encoding WT or mutant NCL isoforms (see also [32]). As shown in Figure 6, panel B, the production of NCL, either full-length (WT), ∆N_275–710_, or the N_ter_ mutants (all containing the NLS sequence), significantly increased the percentage of cells in which TDP-43 G295S was properly localized into the nucleus compared to control cells (co-transfected with plasmid encoding TDP-43-GFP, G295S, and mKate2), in which only 40% of cells showed nuclear TDP-43 G295S. On the contrary, production of the ∆N_300–710_ NCL mutant, lacking the NLS sequence, was not able to restore TDP-43 G295S nuclear localization. However, no NCL mutant isoform was able to reduce the number of cells with TDP-43 G295S inclusions to the same extent observed for the full-length NCL (Figure 6, panel C), consistent with their incapacity to rescue TDP-43-induced toxicity (see Figure 5, left panel).

Collectively taken, the data supported the idea that NCL is involved in the nuclear-cytoplasmic shuttling of TDP-43 through its ability to move between the cell compartments, strictly requiring its nuclear import via the NLS sequence. They also indicated, however, that this was insufficient to fully restore cell viability, suggesting the need for NCL structural integrity and the existence of additional mechanisms for NCL to suppress the TDP-43-dependent cytotoxicity. Because TDP-43 inclusions have been so far directly tied to cell survival, maintaining the native, full-length NCL protein’s structural integrity therefore appears crucial to its curative ability towards TDP-43.

## 3. Discussion

ALS and FTLD represent two distinct neurodegenerative disorders. ALS is predominantly characterized by progressive degeneration of motor neurons, although other neurons are compromised in some ALS cases (e.g., in the fronto-executive circuits, temporal and parietal cortical regions, basal ganglia, and dorsal root ganglia) [48,49,50,51]. FTLD neuropathology is a spectrum of primary neurodegenerative disorders, most of which are characterized by TDP-43 (or tau) inclusions, covering different subgroups, among which FTD is one of the most common clinical manifestations related to FTLD-TDP-43 proteinopathy [52]. FTLD/FTD neuropathology is associated with progressive deficits in language, behavior, and/or cognition. It causes selective degeneration of the frontal and temporal lobes of the brain, primarily resulting in behavioral dysfunction, such as changes in personality and executive function, loss of volition, or language deficits [53,54]. However, there is a significant clinical, genetic, and pathological overlap between ALS and FTD, and it is now thought that they are distinct manifestations of the same neuropathological disease spectrum [55,56].

One of the shared hallmarks of ALS and FTLD is the presence of cytosolic neuronal inclusions whose major component is the ubiquitinated and hyperphosphorylated TDP-43 protein [19]. Since such inclusions were discovered in almost 95% of ALS cases and roughly 50% of FTLD/FTD cases, these pathologies have been designated TDP-43 proteinopathies [57,58]. Moreover, both familial and sporadic ALS and rare cases of FTLD/FTD have been genetically related to mutations in the gene encoding TDP-43 (*TARDBP*), thus linking TDP-43 to the onset of both pathologies [24,56,59]. Generally, TDP-43 is affected by these mutations in a variety of ways, such as an increased propensity to aggregate, abnormal cytoplasmic mislocalization, change in phase transition properties, resistance to proteases, or altered binding to other proteins [5,60]. However, such mechanisms are strictly connected with each other and with TDP-43-related neuronal damage and death. It can indeed be argued that modifications of TDP-43 properties may cause its misplacement and entrapment into inclusions, leading to the loss of normal function of nuclear TDP-43. As a result, TDP-43 dysfunction and aggregation into toxic species can progress in a vicious cycle.

Nevertheless, more studies are needed to unveil the molecular mechanisms underlying the cytotoxicity related to the alteration of TDP-43 structure and functionality. In this context, the yeast *S. cerevisiae* represents the simplest eukaryotic cell model in which it is possible to recapitulate TDP-43-dependent toxicity and to rapidly assay the impact of disease-associated TDP-43 mutations. Accordingly, yeast models recently became extremely useful to identify many genetic/molecular modifiers (i.e., enhancers and/or suppressors) of the neurotoxicity induced by the ectopic expression of sequences encoding for WT or mutant TDP-43 [25,26,27,30,31].

Here, we demonstrated that three different FTLD-related TDP-43 missense mutations are causative of massive death in yeast cells, but—similarly to WT and ALS-linked mutant TDP-43—the cytotoxicity can be efficiently counteracted by the co-expression of vectors encoding for human NCL, further confirming our previous results indicating NCL as a player able to greatly reduce the TDP-43-induced cell damage [32]. Importantly, we also structurally dissected the NCL domains that may be involved in its ability to cure TDP-43 toxicity. In yeast models, data demonstrated that the N-terminal domain of NCL is of primary relevance in mediating the suppression of TDP-43-induced cell death since NCL ΔN_275–710_ and ΔN_300–710_ deletion mutants lost the protective function. However, data also suggested that the protective role of NCL does not rely on the localization of the protein. Firstly, both the above NCL mutants were distributed throughout the cell, similarly to the WT protein, yet they are ineffective; secondly, the NCL ∆C and N_ter_ truncated forms, showing similar distribution with each other but different localization compared to the WT isoform, were either still fully active (NCL ∆C) or completely ineffective (NCL N_ter_) as suppressors of TDP-43-induced toxicity, respectively, further supporting that in yeast cells, no causal relation exists between NCL localization and its safeguarding activity against TDP-43 damage. Furthermore, although in human cells the NCL N-terminal domain is crucial for the nucleolar localization of the protein [61], our data in the HEK293T model confirmed that, despite its nucleolar/nuclear localization, the N-terminal region of NCL was unable to rescue TDP-43 lethality, consistently with observations in yeast cells.

Beyond determining the nucleolar localization, several other roles were attributed to the NCL N-terminal domain. Among such functions, it seems to be necessary, although not sufficient, for NCL histone chaperone activity, with relevant implications in the modulation of chromatin condensation [62]. Hence, it is fair to hypothesize that TDP-43 toxicity in yeast cells might also be related to an impaired expression of some genes (caused by changes in chromatin structures), which can only be corrected by full-length NCL, but not by loss-of-function deleted forms. In addition, it must be considered that NCL roles in chromatin remodeling (via its N-terminal domain) are also implicated in multiple signaling pathways, including the anti-apoptotic effects accredited to the protein.

The observation that the NCL N-terminal domain of NCL is not able by itself to rescue TDP-43-induced toxicity implies that any function attributed to such domain is required, but not sufficient, for the safeguarding role of NCL. Since the ability of the N-terminal domain of NCL to protect yeast cells from TDP-43 toxicity was completely re-established by the addition of the (last) two RRMs (NCL ∆RRM1–2), we can argue that the binding to RNA/DNA is also necessary for NCL suppressive function towards TDP-43 toxicity. Yet it is not sufficient, as the expression of the NCL middle portion only (NCL ∆N/∆C) did not rescue yeast cell survival. Furthermore, the lack of effects of the GAR domain (NCL ∆C) deletion on NCL protective potentiality suggests that this domain is not crucial for this function in yeast cells, although it contributes to NCL subcellular localization in neurons [42].

The results obtained in our yeast models pointed out that, except for the C-terminal GAR, almost the entire sequence of NCL (aa 1–650) is required for the protein to retain its capacity to cure the TDP-43 cytotoxicity, therefore definitively relating this NCL property to both its N-terminal and central (RRMs) domains. The molecular mechanisms leading to the suppression of TDP-43 lethality in yeast cells may thus also involve the ability of NCL to bind both proteins (histones) and nucleic acids.

Similar considerations can be formulated for human cells, where we showed that, irrespective of the presence of the NLS sequence, the protective effect of NCL against TDP-43-induced toxicity was lost after the removal of the N-terminal region. Nevertheless, as observed in the yeast model, the expression of only the N-terminal domain of NCL failed to rescue HEK293T cells from TDP-43 damage, definitively demonstrating that this domain alone was not sufficient for the protective role of NCL. On the other hand, overexpression of NCL mutants carrying the NLS signal sequence promoted the proper localization of TDP-43 in the nuclear compartment, yet—surprisingly—the number of cells with cytosolic TDP-43 inclusions was still much higher than that observed in the presence of full-length NCL.

Collectively taken, these data indicate that NCL is involved in the nuclear–cytoplasmic shuttling of TDP-43, possibly thanks to its ability to move between cell compartments and strictly requiring its nuclear import via the NLS sequence. The data also provided evidence that this is insufficient to restore cell viability, suggesting the existence of additional mechanisms for NCL to antagonize TDP-43 toxicity that could require the structural integrity of the protein. Because TDP-43 inclusions are directly tied to cell survival, maintaining the native, full-length NCL protein’s structural integrity appears to be crucial to totally prevent the accumulation of such inclusions. This result might implicate further mechanisms, which are still unknown yet would entail the ability of NCL to interact with proteins and DNA/RNA, in agreement with what is observed in yeast cells.

In conclusion, additional studies are required to further investigate the relationship between the NCL-mediated suppression of TDP-43 harmfulness and the (multiple) post-translational modifications targeting specific residues of its N-terminal region (e.g., phosphorylation, glycosylation, acetylation). In this context, the use of yeast cell models appears to be a rapid and reliable strategy to evaluate the effects of site-specific NCL mutants on their functional properties, as we have shown in this work.

## 4. Materials and Methods

### 4.1. Strains, Plasmids and Primers

Yeast and bacterial strains used in this study are listed in Supplementary Table S1, and plasmidic vectors are in Supplementary Table S2. Experimental procedures to generate the different plasmids are described in “Supplementary methods”. Primer sequences are reported in Supplementary Table S3.

### 4.2. Yeast Manipulation and Cell Viability Assay

Yeast cells manipulations were performed according to standard protocols [63,64]. Cells were cultivated at 30 °C in either rich medium (10 g/L Bacto-yeast extract, 20 g/L Bacto-peptone) supplemented with 20 g/L of glucose (YPD) or galactose (YPG) as a carbon source, or in synthetic minimal medium (1.7 g/L yeast nitrogen base without amino acids, 5 g/L ammonium sulfate) containing 20 g/L of glucose (SD) or galactose (SG), and the specific nutrients for the selection of transformed yeast cells. Media components were from Difco (Thermo Fischer Scientific, Waltham, MA, USA), and chemical compounds were from Sigma-Aldrich (St. Louis, MO, USA). Yeast cells transformation was performed using the PEG/lithium acetate method [65]. Yeast viability (spot) assay was performed as described [66] by checking the ability of yeast cells to grow and proliferate on solid medium. Briefly, yeast cells were grown overnight at 30 °C in liquid medium, then the cultures were normalized to OD_600_ = 1 and serially diluted, and 5–10 μL drops were spotted on selective solid medium containing either glucose or galactose before finally incubating the plates at 30 °C for different numbers of days, as indicated.

### 4.3. HEK293T Cell Culture, Transfection and Cell Viability Assay

HEK293T cells were grown in Dulbecco’s modified Eagle medium supplemented with 10% (*v*/*v*) fetal bovine serum, 2 mM L-glutamine, 100 U/mL penicillin, and 100 μg/mL streptomycin (37 °C, 5% CO_2_). Twenty-four hours before transfection, HEK293T cells were seeded at 30% of confluence in 13 mm glass coverslips (for confocal imaging), into a 12-multiwell plate (for WB), or in 96-multiwell plate (for MTS assay). Transfection was performed using the Lipofectamine 3000 transfection kit (Invitrogen) following the manufacturer’s instructions and analyzed 48 h after transfection. Generally, we obtained 40% co-transfection efficiency, determined as the ratio between the number of GFP and mKate2 positive cells and the total population (Hoechst stained). Cell viability was assessed as reported [32] using the CellTiter96 Aqueous One 5 Solution Assay (Promega. Madison, WI, USA). After incubation with the MTS reagent (90 min, 37 °C), the absorbance of reduced MTS (λ = 490) was determined using a Microplate Reader (TECAN, Männedorf Switzerland) spectrophotometer.

### 4.4. Confocal Microscopy

To perform confocal microscopy analyses in yeast, the cells were incubated for 24 h in galactose-containing medium and fixed in 4% formaldehyde. After washing, cells were resuspended in 1 M sorbitol, and dropped onto a slide, then covered with a coverslip (Superfrost, BDH) [67]. Similarly, transfected HEK293T human cells were washed twice with phosphate-buffered saline (PBS) and fixed with formaldehyde [3.7% (*v*/*v*) in PBS]. Cell nuclei were counter-stained with Hoechst 33342 [(5 μg/Ml in PBS) 10 min, RT, Sigma-Aldrich] and coverslips were finally washed in PBS and mounted onto microscope slides using a fluorescence mounting medium (DAKO) and observed. Images of both yeast and human cells were finally collected with a Leica SP5 confocal microscope using the 63X HCX PL APO (NA 1.4) oil-immersion objectives. Laser excitation line, power intensity, and emission range were chosen accordingly to fluorophore in different samples to minimize bleed-through. Imaging was performed at 1024 × 1024 pixels, with a 200 Hz acquisition rate, by capturing Z-series that covered the entire field of interest. Z-projection images were obtained using the Fiji/ImageJ software (https://imagej.net/ij/, accessed on 25 November 2023).

### 4.5. Cell Lysis and Protein Extraction

Total proteins were extracted and purified from yeast cells following the trichloroacetic acid (TCA) method [68]. Briefly, 5 Ml of yeast cultures (OD_600_ = 1) were harvested, and cells were resuspended in TCA 20%. Then, 100 Μl of glass beads (Sigma-Aldrich) were added and yeast mechanical lysis was performed via alternating 1 min cycles with a bead-beater (Magnalyzer, Roche, Vienna, Austria) and 1 min on ice. TCA 10% was added, and samples were centrifuged (10 min, 14,000 rpm). Pellet was finally resuspended in 1× Laemmli Sample Buffer with the addition of Tris-HCl pH 8.8. After boiling (98 °C, 5 min), samples were cleared with centrifugation and the proteins in the supernatant were quantified using the Bradford assay.

For human cells, HEK293T were collected 48 h after transfection, washed twice with PBS, and lysed with a buffer containing SDS 2.3% (*v*/*v*), Tris-HCl 62.5 mM pH 6.8, and glycerol 10% (*w*/*v*) (70 μL/well). Cell debris was removed with centrifugation (14,000× *g*, 10 min, 4 °C) and the total protein content was determined using the BCA assay kit (Thermo Fisher Scientific).

### 4.6. Western Blot and Antibodies

A quantity of 20 μg of total proteins were diluted in reducing sample buffer (SDS 2.3% (*v*/*v*), Tris-HCl 62.5 mM pH 6.8, glycerol 10% (*w*/*v*), 50 mM DTT, and 0.01% (*w*/*v*) bromophenol blue) and subjected to SDS-PAGE using 12% (*w*/*v*) acrylamide-N,N’-methylenebisacrylamide (37.5:1 (*w*/*w*)). Proteins were then transferred onto polyvinylidene difluoride (PVDF) membranes (0.45 μm pore size; Bio-Rad Laboratories, Hercules, CA, USA) and blocked through incubation (1 h, RT) with a blocking solution [5% (*w*/*v*) non-fat dry milk (Bio-Rad Laboratories) in TRIS-buffered saline (TBS) with 0.1% (*w*/*v*) Tween-20 (TBS-T)]. Membranes were then probed (overnight, 4 °C) with the desired primary antibody diluted in TBS-T containing 1% (*w*/*v*) bovine serum albumin (BSA). After three washings with TBS-T, membranes were incubated (1 h, RT) with horseradish peroxidase-conjugated anti-mouse or anti-rabbit IgG secondary antibody (Sigma-Aldrich, cat. nos. A9044 and A0545, respectively) (1:60,000 in blocking solution), depending on the primary antibody. Immunoreactive bands were visualized using an enhanced chemiluminescence reagent kit (EMD Millipore, Darmstadt, Germany) and digitalized by means of a UVItec imaging system (Eppendorf, Hamburg, Germany). To check for even protein loading, membranes were stained with Coomassie brilliant blue (Sigma-Aldrich).

The following primary antibodies (Abs) were used (dilutions in parentheses): anti-TDP-43 mouse monoclonal (m)Ab (1:1000, Santa Cruz Biotechnology, Dallas, Texas, USA, #sc-376532); anti RFP mouse mAb (1:5000, Abcam, Cambridge, UK, #62341); anti GAPDH (1:2000, Cell Signaling Danvers, MA, USA, #2118).

### 4.7. Statistical Analysis

Data were analyzed with using GraphPAD (version 8.0.1) software. Statistics were performed as indicated in the figure legends. Briefly, to compare three or more groups, the parametric one-way Anova or the non-parametric Kruskal–Wallis test were used, depending on whether the data distribution was normal or not (data did not pass the test of normality, *p* > 0.05), respectively. Data reported in graphs represent mean ± standard error of mean. The significance level was set at *p* < 0.05. n, reported in the legends, indicates that data were derived from at least three independent experiments. HEK293T cells containing nuclear TDP-43 were manually counted at 3D presentation at maximum projection mode using ImageJ software (https://imagej.net/ij/, accessed on 25 November 2023) by considering cells in which the blue (nuclear staining) and green (GFP) signals were overlapped. This number was divided by the total number of co-transfected cells (in which the red (mKate2) and green (GFP) signals were present). Cells containing TDP-43 foci were manually counted as described above and divided by the manually counted number of cells co-expressing both TDP-43 and either mKate2 or NCL isoforms (positive both to green and red signals). Both analyses were performed in ten different fields of three independent co-transfections, for a total of 30 fields for each condition.

## Data Availability

The data presented in this study are available on request from the corresponding authors.

## Supplementary Materials

The following supporting information can be downloaded at: https://www.mdpi.com/article/10.3390/ijms242417466/s1.

## References

[1] Buratti E., Baralle F.E. (2001). Characterization and Functional Implications of the RNA Binding Properties of Nuclear Factor TDP-43, a Novel Splicing Regulator OfCFTR Exon 9. J. Biol. Chem..

[2] Ayala Y.M., Zago P., D’Ambrogio A., Xu Y.-F., Petrucelli L., Buratti E., Baralle F.E. (2008). Structural Determinants of the Cellular Localization and Shuttling of TDP-43. J. Cell Sci..

[3] Ou S.H., Wu F., Harrich D., García-Martínez L.F., Gaynor R.B. (1995). Cloning and Characterization of a Novel Cellular Protein, TDP-43, That Binds to Human Immunodeficiency Virus Type 1 TAR DNA Sequence Motifs. J. Virol..

[4] Ling S.-C., Polymenidou M., Cleveland D.W. (2013). Converging Mechanisms in ALS and FTD: Disrupted RNA and Protein Homeostasis. Neuron.

[5] Prasad A., Bharathi V., Sivalingam V., Girdhar A., Patel B.K. (2019). Molecular Mechanisms of TDP-43 Misfolding and Pathology in Amyotrophic Lateral Sclerosis. Front. Mol. Neurosci..

[6] Buratti E., Baralle F.E. (2012). TDP-43: Gumming up Neurons through Protein-Protein and Protein-RNA Interactions. Trends Biochem. Sci..

[7] Ayala Y.M., De Conti L., Avendaño-Vázquez S.E., Dhir A., Romano M., D’Ambrogio A., Tollervey J., Ule J., Baralle M., Buratti E. (2011). TDP-43 Regulates Its MRNA Levels through a Negative Feedback Loop. EMBO J..

[8] Afroz T., Hock E.-M., Ernst P., Foglieni C., Jambeau M., Gilhespy L.A.B., Laferriere F., Maniecka Z., Plückthun A., Mittl P. (2017). Functional and Dynamic Polymerization of the ALS-Linked Protein TDP-43 Antagonizes Its Pathologic Aggregation. Nat. Commun..

[9] Cushman M., Johnson B.S., King O.D., Gitler A.D., Shorter J. (2010). Prion-like Disorders: Blurring the Divide between Transmissibility and Infectivity. J. Cell Sci..

[10] Fuentealba R.A., Udan M., Bell S., Wegorzewska I., Shao J., Diamond M.I., Weihl C.C., Baloh R.H. (2010). Interaction with Polyglutamine Aggregates Reveals a Q/N-Rich Domain in TDP-43. J. Biol. Chem..

[11] Ayala Y.M., Pantano S., D’Ambrogio A., Buratti E., Brindisi A., Marchetti C., Romano M., Baralle F.E. (2005). Human, Drosophila, and C. Elegans TDP43: Nucleic Acid Binding Properties and Splicing Regulatory Function. J. Mol. Biol..

[12] Buratti E., Brindisi A., Giombi M., Tisminetzky S., Ayala Y.M., Baralle F.E. (2005). TDP-43 Binds Heterogeneous Nuclear Ribonucleoprotein A/B through Its C-Terminal Tail. J. Biol. Chem..

[13] Freibaum B.D., Chitta R.K., High A.A., Taylor J.P. (2010). Global Analysis of TDP-43 Interacting Proteins Reveals Strong Association with RNA Splicing and Translation Machinery. J. Proteome Res..

[14] Conicella A.E., Zerze G.H., Mittal J., Fawzi N.L. (2016). ALS Mutations Disrupt Phase Separation Mediated by α-Helical Structure in the TDP-43 Low-Complexity C-Terminal Domain. Structure.

[15] Guo W., Chen Y., Zhou X., Kar A., Ray P., Chen X., Rao E.J., Yang M., Ye H., Zhu L. (2011). An ALS-Associated Mutation Affecting TDP-43 Enhances Protein Aggregation, Fibril Formation and Neurotoxicity. Nat. Struct. Mol. Biol..

[16] Budini M., Romano V., Quadri Z., Buratti E., Baralle F.E. (2015). TDP-43 Loss of Cellular Function through Aggregation Requires Additional Structural Determinants beyond Its C-Terminal Q/N Prion-like Domain. Hum. Mol. Genet..

[17] Mompeán M., Buratti E., Guarnaccia C., Brito R.M.M., Chakrabartty A., Baralle F.E., Laurents D.V. (2014). Structural Characterization of the Minimal Segment of TDP-43 Competent for Aggregation. Arch. Biochem. Biophys..

[18] Jiang L.-L., Che M.-X., Zhao J., Zhou C.-J., Xie M.-Y., Li H.-Y., He J.-H., Hu H.-Y. (2013). Structural Transformation of the Amyloidogenic Core Region of TDP-43 Protein Initiates Its Aggregation and Cytoplasmic Inclusion. J. Biol. Chem..

[19] Neumann M., Sampathu D.M., Kwong L.K., Truax A.C., Micsenyi M.C., Chou T.T., Bruce J., Schuck T., Grossman M., Clark C.M. (2006). Ubiquitinated TDP-43 in Frontotemporal Lobar Degeneration and Amyotrophic Lateral Sclerosis. Science.

[20] Scotter E.L., Chen H.J., Shaw C.E. (2015). TDP-43 Proteinopathy and ALS: Insights into Disease Mechanisms and Therapeutic Targets. Neurotherapeutics.

[21] Arai T., Hasegawa M., Akiyama H., Ikeda K., Nonaka T., Mori H., Mann D., Tsuchiya K., Yoshida M., Hashizume Y. (2006). TDP-43 Is a Component of Ubiquitin-Positive Tau-Negative Inclusions in Frontotemporal Lobar Degeneration and Amyotrophic Lateral Sclerosis. Biochem. Biophys. Res. Commun..

[22] Boeve B.F., Boxer A.L., Kumfor F., Pijnenburg Y., Rohrer J.D. (2022). Advances and Controversies in Frontotemporal Dementia: Diagnosis, Biomarkers, and Therapeutic Considerations. Lancet Neurol..

[23] Amador-Ortiz C., Lin W.-L., Ahmed Z., Personett D., Davies P., Duara R., Graff-Radford N.R., Hutton M.L., Dickson D.W. (2007). TDP-43 Immunoreactivity in Hippocampal Sclerosis and Alzheimer’s Disease. Ann. Neurol..

[24] Sreedharan J., Blair I.P., Tripathi V.B., Hu X., Vance C., Rogelj B., Ackerley S., Durnall J.C., Williams K.L., Buratti E. (2008). TDP-43 Mutations in Familial and Sporadic Amyotrophic Lateral Sclerosis. Science.

[25] Johnson B.S., Snead D., Lee J.J., McCaffery J.M., Shorter J., Gitler A.D. (2009). TDP-43 Is Intrinsically Aggregation-Prone, and Amyotrophic Lateral Sclerosis-Linked Mutations Accelerate Aggregation and Increase Toxicity. J. Biol. Chem..

[26] Armakola M., Hart M.P., Gitler A.D. (2011). TDP-43 Toxicity in Yeast. Methods.

[27] Johnson B.S., McCaffery J.M., Lindquist S., Gitler A.D. (2008). A Yeast TDP-43 Proteinopathy Model: Exploring the Molecular Determinants of TDP-43 Aggregation and Cellular Toxicity. Proc. Natl. Acad. Sci. USA.

[28] Kryndushkin D., Wickner R.B., Shewmaker F. (2011). FUS/TLS Forms Cytoplasmic Aggregates, Inhibits Cell Growth and Interacts with TDP-43 in a Yeast Model of Amyotrophic Lateral Sclerosis. Protein Cell.

[29] Park S.K.S., Park S.K.S., Liebman S.W. (2019). Respiration Enhances TDP-43 Toxicity, but TDP-43 Retains Some Toxicity in the Absence of Respiration. J. Mol. Biol..

[30] Park S.-K., Hong J.Y., Arslan F., Kanneganti V., Patel B., Tietsort A., Tank E.M.H., Li X., Barmada S.J., Liebman S.W. (2017). Overexpression of the Essential Sis1 Chaperone Reduces TDP-43 Effects on Toxicity and Proteolysis. PLoS Genet..

[31] Park S.-K., Park S., Liebman S.W. (2022). TDP-43 Toxicity in Yeast Is Associated with a Reduction in Autophagy, and Deletions of TIP41 and PBP1 Counteract These Effects. Viruses.

[32] Peggion C., Massimino M.L., Stella R., Bortolotto R., Agostini J., Maldi A., Sartori G., Tonello F., Bertoli A., Lopreiato R. (2021). Nucleolin Rescues TDP-43 Toxicity in Yeast and Human Cell Models. Front. Cell. Neurosci..

[33] Mongelard F., Bouvet P. (2007). Nucleolin: A MultiFACeTed Protein. Trends Cell Biol..

[34] Tajrishi M.M., Tuteja R., Tuteja N. (2011). Nucleolin. Commun. Integr. Biol..

[35] Ginisty H., Sicard H., Roger B., Bouvet P. (1999). Structure and Functions of Nucleolin. J. Cell Sci..

[36] Jia W., Yao Z., Zhao J., Guan Q., Gao L. (2017). New Perspectives of Physiological and Pathological Functions of Nucleolin (NCL). Life Sci..

[37] Tonello F., Massimino M.L., Peggion C. (2022). Nucleolin: A Cell Portal for Viruses, Bacteria, and Toxins. Cell. Mol. Life Sci..

[38] Storck S., Thiry M., Bouvet P. (2009). Conditional Knockout of Nucleolin in DT40 Cells Reveals the Functional Redundancy of Its RNA-Binding Domains. Biol. Cell.

[39] Creancier L., Prats H., Zanibellato C., Amalric F., Bugler B. (1993). Determination of the Functional Domains Involved in Nucleolar Targeting of Nucleolin. Mol. Biol. Cell.

[40] Schmidt-Zachmann M.S., Nigg E.A. (1993). Protein Localization to the Nucleolus: A Search for Targeting Domains in Nucleolin. J. Cell Sci..

[41] Pellar G.J., DiMario P.J. (2003). Deletion and Site-Specific Mutagenesis of Nucleolin’s Carboxy GAR Domain. Chromosoma.

[42] Doron-Mandel E., Koppel I., Abraham O., Rishal I., Smith T.P., Buchanan C.N., Sahoo P.K., Kadlec J., Oses-Prieto J.A., Kawaguchi R. (2021). The Glycine Arginine-rich Domain of the RNA-binding Protein Nucleolin Regulates Its Subcellular Localization. EMBO J..

[43] Borroni B., Archetti S., Del Bo R., Papetti A., Buratti E., Bonvicini C., Agosti C., Cosseddu M., Turla M., Di Lorenzo D. (2010). *TARDBP* Mutations in Frontotemporal Lobar Degeneration: Frequency, Clinical Features, and Disease Course. Rejuvenation Res..

[44] Caroppo P., Camuzat A., Guillot-Noel L., Thomas-Antérion C., Couratier P., Wong T.H., Teichmann M., Golfier V., Auriacombe S., Belliard S. (2016). Defining the Spectrum of Frontotemporal Dementias Associated with *TARDBP* Mutations. Neurol. Genet..

[45] Winton M.J., Van Deerlin V.M., Kwong L.K., Yuan W., Wood E.M., Yu C.-E., Schellenberg G.D., Rademakers R., Caselli R., Karydas A. (2008). A90V TDP-43 Variant Results in the Aberrant Localization of TDP-43 in Vitro. FEBS Lett..

[46] Carpentier M., Morelle W., Coddeville B., Pons A., Masson M., Mazurier J., Legrand D. (2005). Nucleolin Undergoes Partial N- and O-Glycosylations in the Extranuclear Cell Compartment. Biochemistry.

[47] Hoja-Łukowicz D., Kedracka-Krok S., Duda W., Lityńska A. (2014). The Lectin-Binding Pattern of Nucleolin and Its Interaction with Endogenous Galectin-3. Cell. Mol. Biol. Lett..

[48] Castelnovo V., Canu E., De Mattei F., Filippi M., Agosta F. (2023). Basal Ganglia Alterations in Amyotrophic Lateral Sclerosis. Front. Neurosci..

[49] Machts J., Loewe K., Kaufmann J., Jakubiczka S., Abdulla S., Petri S., Dengler R., Heinze H.-J., Vielhaber S., Schoenfeld M.A. (2015). Basal Ganglia Pathology in ALS Is Associated with Neuropsychological Deficits. Neurology.

[50] Bede P., Iyer P.M., Finegan E., Omer T., Hardiman O. (2017). Virtual Brain Biopsies in Amyotrophic Lateral Sclerosis: Diagnostic Classification Based on in Vivo Pathological Patterns. Neuroimage Clin..

[51] Riancho J., Paz-Fajardo L., López de Munaín A. (2021). Clinical and Preclinical Evidence of Somatosensory Involvement in Amyotrophic Lateral Sclerosis. Br. J. Pharmacol..

[52] Grossman M., Seeley W.W., Boxer A.L., Hillis A.E., Knopman D.S., Ljubenov P.A., Miller B., Piguet O., Rademakers R., Whitwell J.L. (2023). Frontotemporal Lobar Degeneration. Nat. Rev. Dis. Primers.

[53] Younes K., Miller B.L. (2020). Frontotemporal Dementia: Neuropathology, Genetics, Neuroimaging, and Treatments. Psychiatr. Clin. N. Am..

[54] Bang J., Spina S., Miller B.L. (2015). Frontotemporal Dementia. Lancet.

[55] Couratier P., Corcia P., Lautrette G., Nicol M., Marin B. (2017). ALS and Frontotemporal Dementia Belong to a Common Disease Spectrum. Rev. Neurol..

[56] Kirola L., Mukherjee A., Mutsuddi M. (2022). Recent Updates on the Genetics of Amyotrophic Lateral Sclerosis and Frontotemporal Dementia. Mol. Neurobiol..

[57] Liao Y.-Z., Ma J., Dou J.-Z. (2022). The Role of TDP-43 in Neurodegenerative Disease. Mol. Neurobiol..

[58] Cohen T.J., Lee V.M.Y., Trojanowski J.Q. (2011). TDP-43 Functions and Pathogenic Mechanisms Implicated in TDP-43 Proteinopathies. Trends Mol. Med..

[59] Gendron T.F., Rademakers R., Petrucelli L. (2013). TARDBP Mutation Analysis in TDP-43 Proteinopathies and Deciphering the Toxicity of Mutant TDP-43. J. Alzheimer’s Dis..

[60] Buratti E., Friedmann T., Dunlap J.C., Goodwin S.F.B.T.-A.G. (2015). Chapter One—Functional Significance of TDP-43 Mutations in Disease.

[61] Fujiwara Y., Fujiwara K., Goda N., Iwaya N., Tenno T., Shirakawa M., Hiroaki H. (2011). Structure and Function of the N-Terminal Nucleolin Binding Domain of Nuclear Valosin-Containing Protein-like 2 (NVL2) Harboring a Nucleolar Localization Signal. J. Biol. Chem..

[62] Wise J.F., Berkova Z., Mathur R., Zhu H., Braun F.K., Tao R.-H., Sabichi A.L., Ao X., Maeng H., Samaniego F. (2013). Nucleolin Inhibits Fas Ligand Binding and Suppresses Fas-Mediated Apoptosis in Vivo via a Surface Nucleolin-Fas Complex. Blood.

[63] Amberg D.C., Burke D., Strathern J.N., Burke D., Laboratory C.S.H. (2005). Methods in Yeast Genetics: A Cold Spring Harbor Laboratory Course Manual.

[64] Vokes M., Carpenter A., Ausubel F.M., Brent R., Kingston R.E., David D., Moore D.D., Seidman J.G., Smith J.A., Struhl K. (2008). Current Protocols in Molecular Biology.

[65] Gietz R.D., Woods R.A. (2006). Yeast Transformation by the LiAc/SS Carrier DNA/PEG Method. Methods Mol. Biol..

[66] Mirisola M.G., Braun R.J., Petranovic D. (2014). Approaches to Study Yeast Cell Aging and Death. FEMS Yeast Res..

[67] Noree C., Sirinonthanawech N. (2020). Nuclear Targeted Saccharomyces Cerevisiae Asparagine Synthetases Associate with the Mitotic Spindle Regardless of Their Enzymatic Activity. PLoS ONE.

[68] Wright A.P.H., Bruns M., Hartley B.S. (1989). Extraction and Rapid Inactivation of Proteins FromSaccharomyces Cerevisiae by Trichloroacetic Acid Precipitation. Yeast.

