# Supplementary material for: Structural Integrity of Nucleolin Is Required to Suppress TDP-43-Mediated Cytotoxicity in Yeast and Human Cell Models
Source: Int J Mol Sci. 2023 Dec 14;24(24):17466. doi: 10.3390/ijms242417466 (PMC10744044; doi:10.3390/ijms242417466)
Supplement: Supplementary file 1 [file ijms-24-17466-s001.zip › ijms-2671233-supplementary.pdf]

Supplementary Methods

Yeast strains.
S. cerevisiae strain CEN.PK IMX672 (Euroscarf) and its derivative TDP 2C, carrying 2 copies of the P_{GAL1}-TDP-43-GFP-$Cyc1_{ter}$ expression cassette in the *LEU2* and *HIS3* genomic loci (**Supplementary Table S1**), were used as background strains for all experimental manipulations.

Plasmids propagation.
E. coli strains StellaR (Clontech) and Top10 (Invitrogen) were used as host for cloning procedures and plasmid propagation (**Supplementary Table S1**). *E. coli* cells were grown at 37°C in liquid o r solid (by adding agar 2% (w/v)) Luria-Bertani (LB) medium (Tryptone 1% (w/v), Yeast extract 0.5% (w/v), NaCl 1% (w/v)) containing 100 µg/ml ampicillin (Sigma-Aldrich) for the selection of transform ants.

Plasmids construction
Plasmids to express in yeast the TDP-43 protein, carrying the FTD-related missense mutations A90V, G295S and M359V (**Supplementary Table S2**), were generated by the QuikChange Site-Di rected Mutagenesis kit (Stratagene) using the pRS246GalTDP43-GFP (wild-type) as template . Mutagenic oligonucleotides (**Supplementary Table S3**) were designed using the QuikCh ange Primer Design Program available online on Stratagene website.
Yeast plasmids expressing NCL mutant isoforms were generated using the In-Fusion HD Cloning Kit (Takara-bio). Briefly, PCR products (NCL-derived) (obtained using primers indicated in **Supplementary Table S3**) and the vector (mKate2-pYES2<HIS3> cleaved by BamHI) were purified using the GenElute™ PCR Clean-Up Kit (Sigma-Aldrich) and subjected to In-Fusion Cloning Procedu re following the manufacturer's instruction. Stellar *E. coli* competent cells were then transformed with the In-Fusion reaction mixture. Recombinant plasmids were isolated onto selective LB pla tes and verified both by restriction digestion and by final sequencing. All primers were designed to obtain PCR products with 15 base pairs complementary extensions necessary for the *in vitro* recombination event, according to the Primer Design Tool available online on Takara website.
To overexpress both TDP-43 and NCL proteins in mammalian cells human cells, the insert coding fo r either TDP-43-GFP (WT and G295S), or the NCL-mKate2 isoforms (WT, N_{ter}, $\Delta N_{275-710}$, $\Delta N_{300-710}$), were cleaved from the pYES2<HIS3> plasmids using the BamHI-XhoI, or KpnI-XhoI enzymes, respectively and inserted into the linearized pcDNA3 vectors by using the same enzymes, by *in vitro* DNA ligation. After verification by sequencing, final plasmids were used in experimental assa ys (**Supplementary Table S2**).

***Supplementary Table S1**– Yeast and Bacterial strains.*

Name	Relevant genotype	Origin
S.cerevisiae yeast strains		
IMX672	*MATa; ura3-52; trp1-289; leu2-3,112; his3Δ; can1Δ::cas9-natNT2*	Mans et al. 2015
TDP 2C	*MATa; ura3-52; trp1Δ::*P_{GAL1}-TDP-43-GFP-$Cyc1_{ter}$*; leu2-3,112; his3Δ::*P_{GAL1}-TDP-43-GFP-$Cyc1_{ter}$*; can1Δ::cas9-natNT2*	Peggion, 2021
E.coli bacterial strains		
StellaR	*F-; endA1; supE44; thi-1; recA1; relA1; gyrA96; phoA; Φ80d; lacZΔ M15; Δ(lacZYA-argF) U169; Δ(mrr-hsdRMS-mcrBC); ΔmcrA; λ-*	*Clontech*
Top10	*F-; mcrA Δ(mrr-hsdRMS-mcrBC); ϕ80; lacZΔ M15; ΔlacX74; recA1; araD139; Δ(ara-leu) 7697; galU galK rpsL (StrR); endA1; nupG; λ-*	*Invitrogen*

***Supplementary Table S2** - Plasmids used in this work.*

Name	Origin
S. cerevisiae Plasmids	
mKate-pYES2<His3>	Peggion, 2021
NCL WT-mKate-pYES2<His3>	Peggion, 2021
NCL Nter-mKate2-pYES2<His3>	This paper
NCL $\Delta N_{275-710}$-mKate2-pYES2<His3>	This paper
NCL $\Delta N_{300-710}$-mKate2-pYES2<His3>	This paper
NCL ΔC-mKate2-pYES2<His3>	This paper
NCL ΔN/ΔC-mKate2-pYES2<His3>	This paper
NCL ΔRRM1/2-mKate2-pYES2<His3>	This paper
pRS426 Gal TDP43 GFP	ADDGENE #27467
pRS426 Gal TDP-43 GFP A90V	This paper
pRS426 Gal TDP-43 GFP M359V	This paper
pRS426 Gal TDP-43 GFP G295S	This paper
Mammalian Plasmids	
mKate2-pcDNA3.1	(De Mario et al., 2016)
TDP43-GFP-pcDNA3	This paper
TDP43(G295S)-GFP-pcDNA3	This paper
NCL WT-mKate-pcDNA3	Peggion, 2021
NCL Nter-mKate2-pcDNA3	This paper
NCL $\Delta N_{275-710}$-mKate2-pcDNA3	This paper
NCL $\Delta N_{300-710}$-mKate2-pcDNA3	This paper

Supplementary Table S3 *- Primers.*

Primer Name	Sequence 5'-3'	Use
NCL 5' for	TACCGAGCTCGGATCCATGGTGAAGCTCGCGAAGG	Amplification of NCL (Nter; ΔC)
NCL 3' Rev	CACCATCGATGGATCCCCTTCAAACTTCGTCTTCTTTCC	Amplification of NCL ($\Delta N_{275-710}$; $\Delta N_{300-710}$)
Nter rev	CACCATCGATGGATCCCCGCCTTCCACTTTCTGTTTCTTG GC	Amplification of NCL Nter
$\Delta N_{275-710}$ for	TACCGAGCTCGGATCCATGGAAGCACCTGGAAAACGAA AGAAGG	Amplification of NCL $\Delta N_{275-710}$
$\Delta N_{300-710}$ for	TACCGAGCTCGGATCCATGGGCACAGAACCGACTACGG C	Amplification of NCL $\Delta N_{300-710}$
ΔC rev	CACCATCGATGGATCCCCTTCACCCTTAGGTTTGGCCCAG TCC	Amplification of NCL (ΔN/ΔC; ΔC)
ΔRRM1/2 for	AAGTGGAACAAGACTATAGAGGTGGAAAGAA	Deletion of NCL RRM1/2 (Δ300–471)
ΔRRM1/2 rev	AGTCTTGTTCCACTTTCTGTTTCTTGGCTT	Deletion of NCL RRM1/2 (Δ300–471)
TDP A90V for	ACAGATGTCTCATCAGCAGTGAAAGTGAAAAGA	TDP-43 site-directed A90V mutagenesis
TDP A90V rev	TGATGAGACATCTGTCTCATCCATTTTTCTTTT	TDP-43 site-directed A90V mutagenesis
TDP G295S for	CAGAGGGTCTGGAGCTGGTTTGGGAAACAATC	TDP-43 site-directed G295S mutagenesis
TDP G295S rev	GCTCCAGACCCTCTGCTATTACCAAATCCACCC	TDP-43 site-directed G295S mutagenesis
TDP M359V for	AGGCAACGTACAGAGGGAGCCAAACCAGG	TDP-43 site-directed M359V mutagenesis
TDP M359V rev	CTCTGTACGTTGCCTTGGTTTTGGTTATTACCC	TDP-43 site-directed M359V mutagenesis

Supplementary Figures

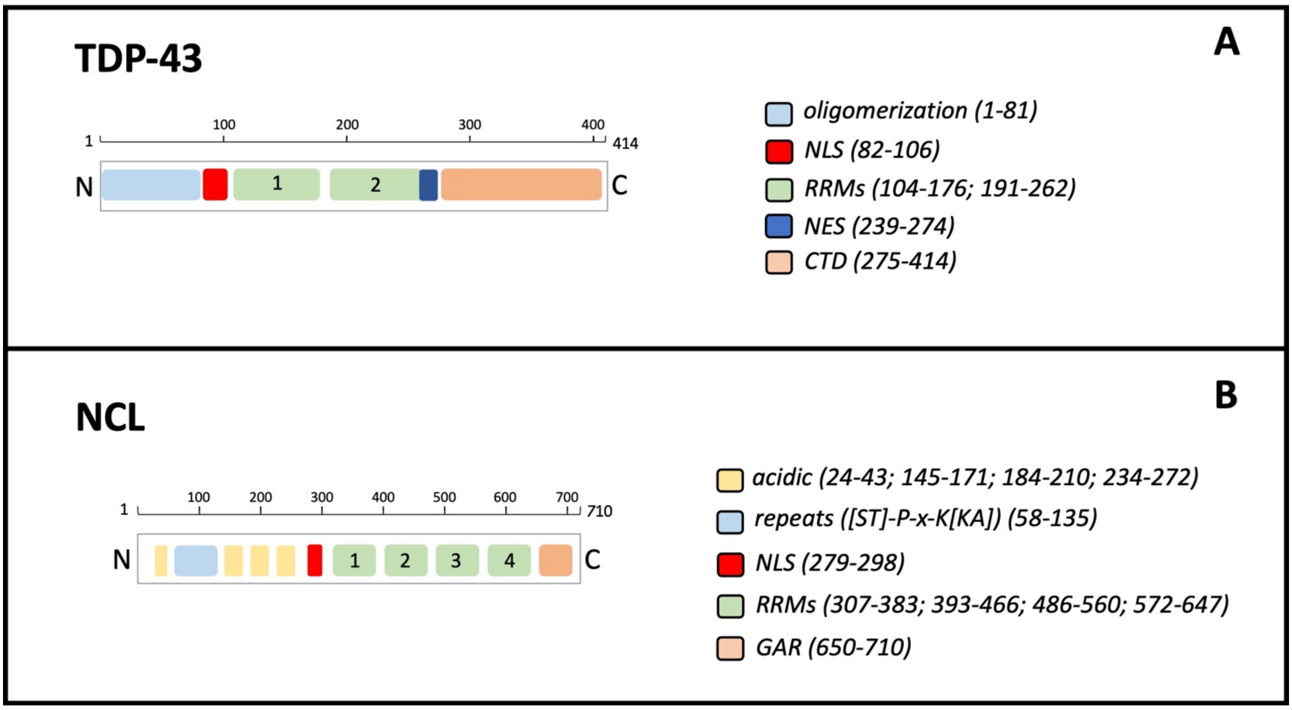

Supplementary Figure S1. A) Structure of human TDP-43 protein. Structural elements of TDP-43 sequence are indicated by different colors: the globular N-terminal domain (implicated in self-oligomerization), the Nuclear Localization Signal (NLS), the 2 tandem RNA-recognition motifs (RRM1-2) (required to bind nuclear transcripts), the Nuclear Export Signal (NES), and the glycine-rich C-terminal domain (CTD) (associated with prion-like properties).
B) Structure of human NCL protein. As above, structural NCL elements are indicated by different colors: the N-terminal acidic aa stretches (necessary to the interaction with basic proteins such as histones); the tandem repeats ([ST]-P-x-K[KA]) (possibly targeted by proline-directed kinases); the Nuclear Localization Sequence (NLS); the 4 RRM domains (required to specific interactions with mRNA and rRNA); the C-terminal disordered domain rich in Glycine and Alanine residues (GAR).

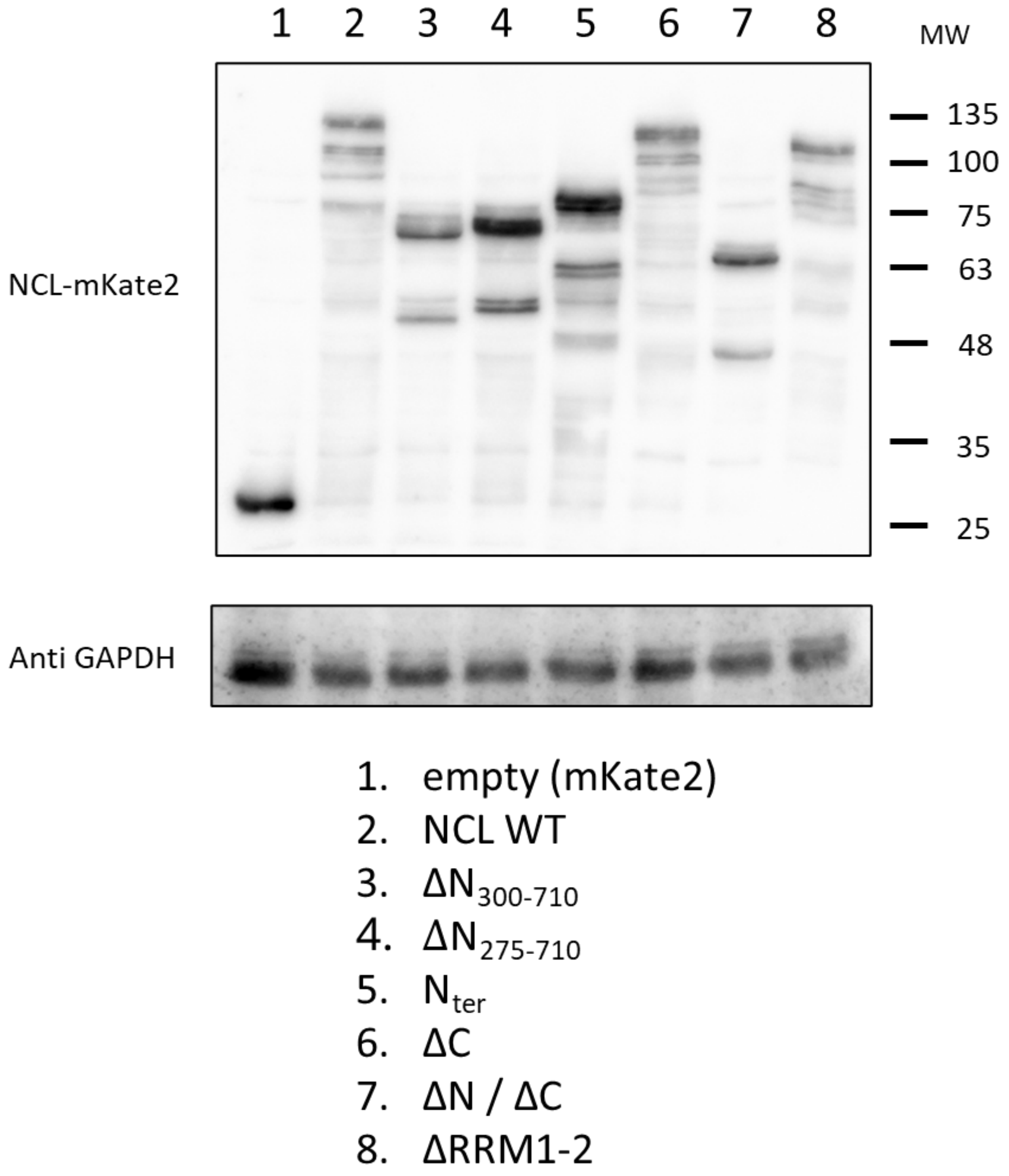

Supplementary Figure S2. Wild-type (WT) and the indicated NCL mutant isoforms are efficiently expressed in yeast cells as mKate2-fusion proteins. After 24 h of induction by growth in a galactose-containing medium, total proteins were extracted from CEN.PK cells transformed with the different NCL plasmids (or empty vector) and analysed by Western Blot (WB) using anti-RFP (upper panel) and anti-GAPDH (lower panel) antibodies. Similar results were obtained in three independent experiments.

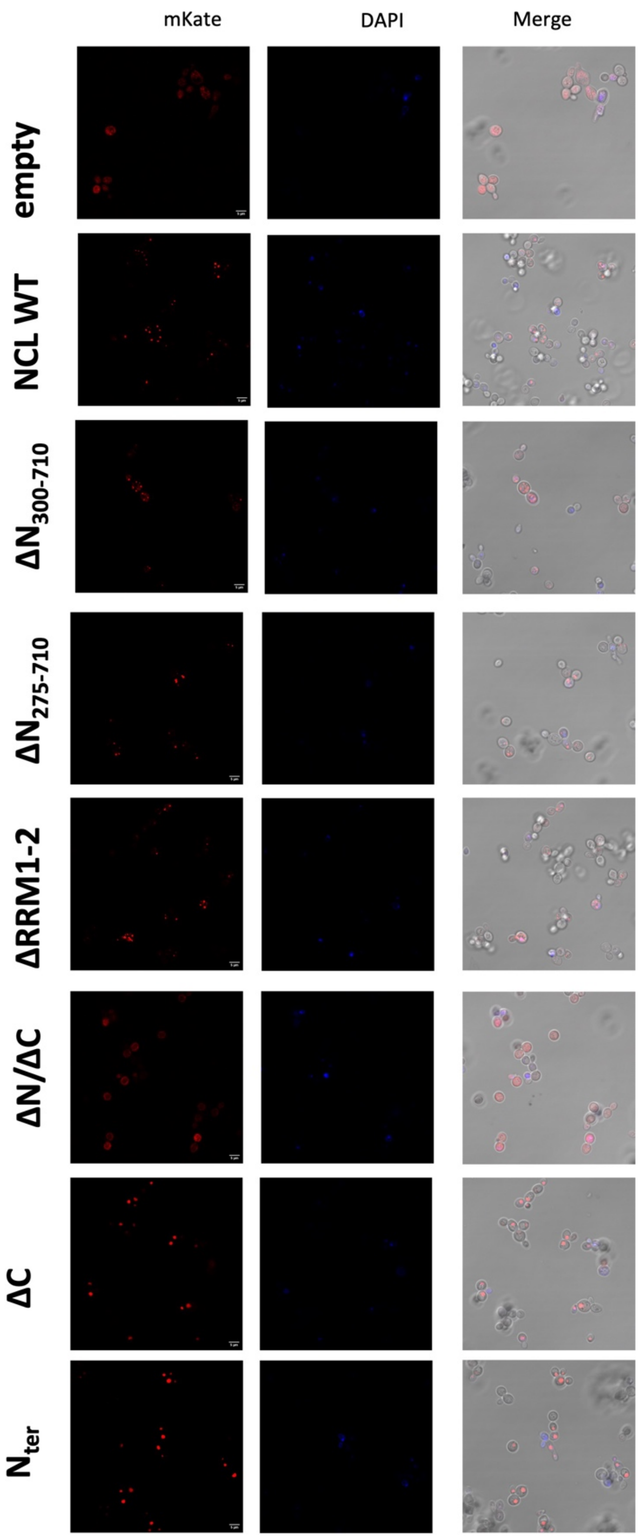

Supplementary Figure S3. Representative images of yeast CEN.PK cells transformed with plasmids expressing either WT or mutant NCL isoforms fused to mKate2 (or mKate2 alone as control,"empty"), and stained with the nuclear marker DAPI. Merged images also include the corresponding differential interference contrast microphotographs. Scale bar are indicated (5 μm).

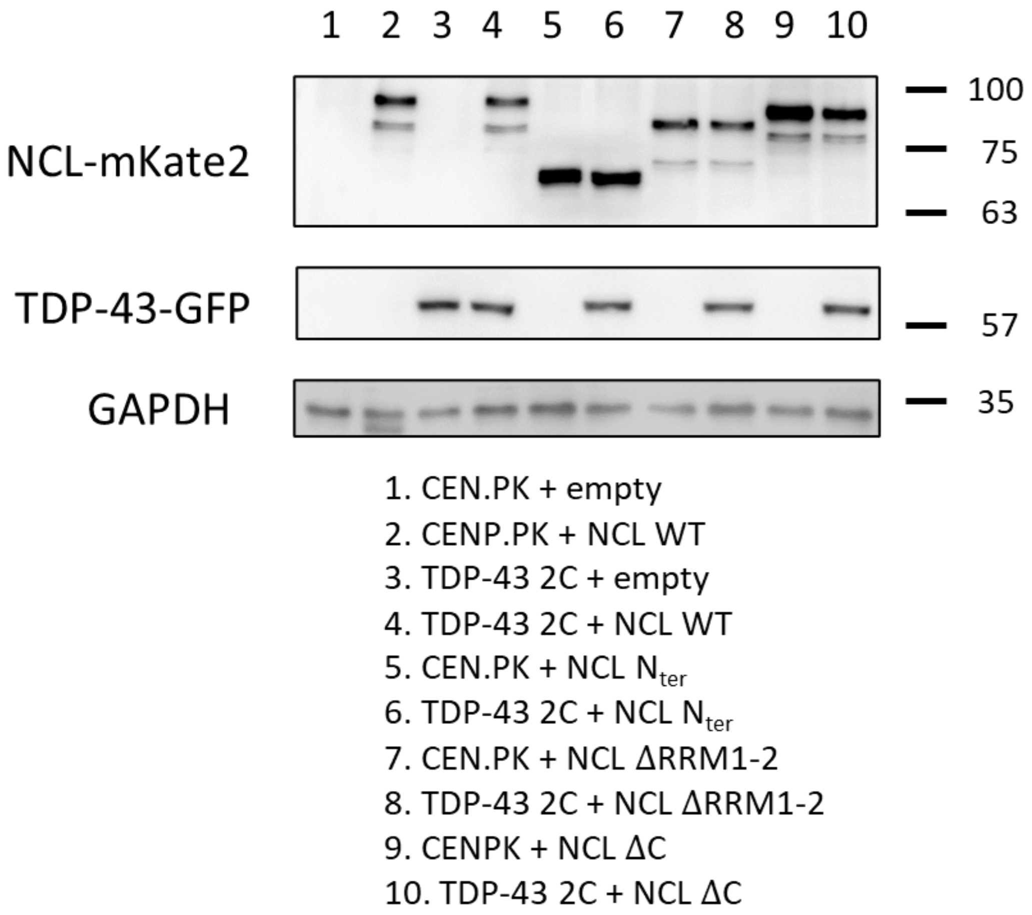

Supplementary Figure S4. Co-expression of either wild-type (WT) or the indicated NCL mutant isoforms does not perturb the expression of TDP-43 in yeast cells. After 24 h of induction by incubation in a galactose-containing medium, total proteins were extracted from either CEN.PK, or CEN.PK TDP-43-GFP 2C cells, transformed with the different NCL plasmids (or empty vector), and subjected to WB analysis using anti-RFP (upper panel), anti-GFP (middle panel), and anti-GAPDH (lower panel) antibodies. Similar results were obtained in three independent experiments.

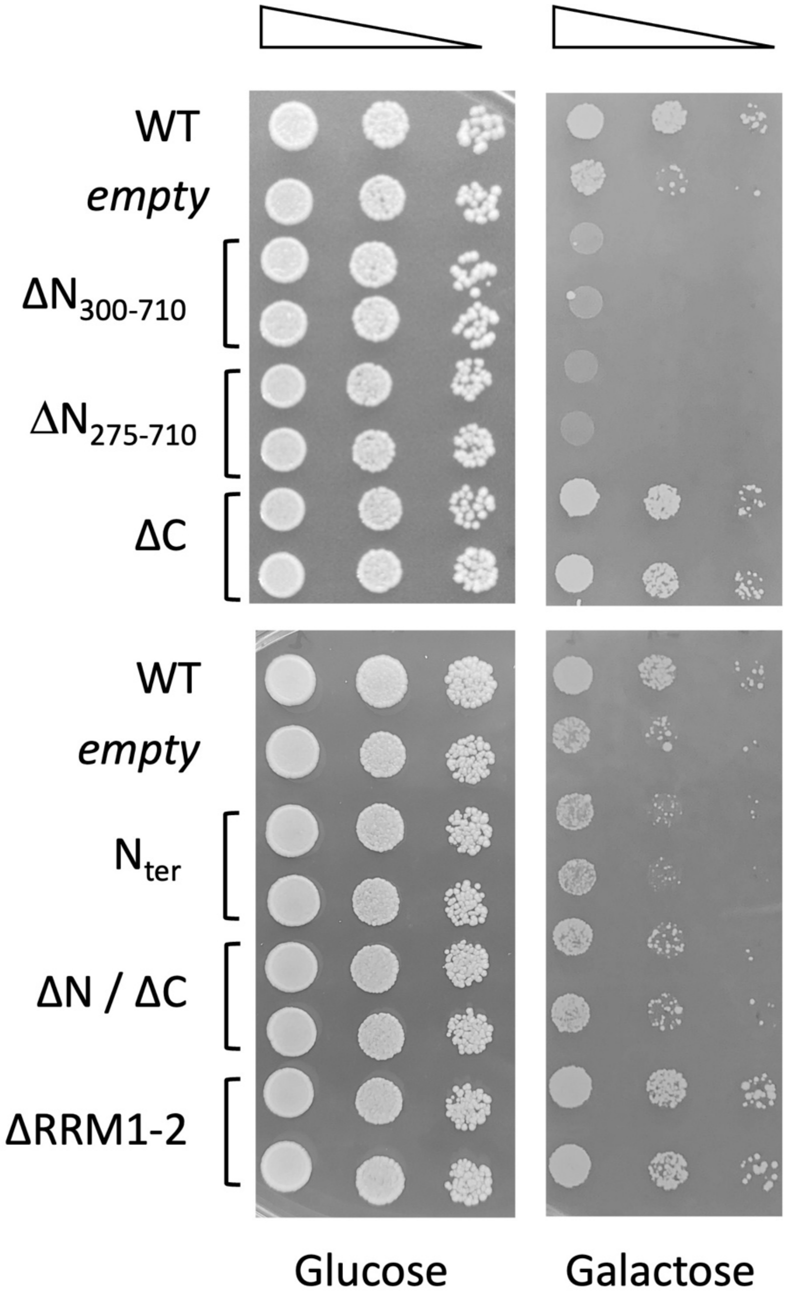

Supplementary Figure S5. Effects of the NCL mutations on its ability to alleviate the TDP-43-dependent cytotoxicity in yeast cells. Growth assay of *S. cerevisiae* CEN.PK strain co-transformed with the multi-copy plasmid expressing TDP-43 (pRS426Gal-TDP43-GFP) and the NCL-mKate2-pYES2-derived plasmids encoding for either NCL wild-type (WT) or the indicated mutant isoforms. Cells carrying the TDP-43 plasmid and transformed with the empty vector (mKate2-pYES2) were considered as control. Yeast cells (OD_{600} = 1) of each strain were serially diluted (10-fold) and spotted onto either repressing condition as control (glucose), or in inducing medium plates (galactose) and incubated at 30°C for 3 (glucose) or 5 (galactose) days. Data are representative of three independent experiments with similar results.

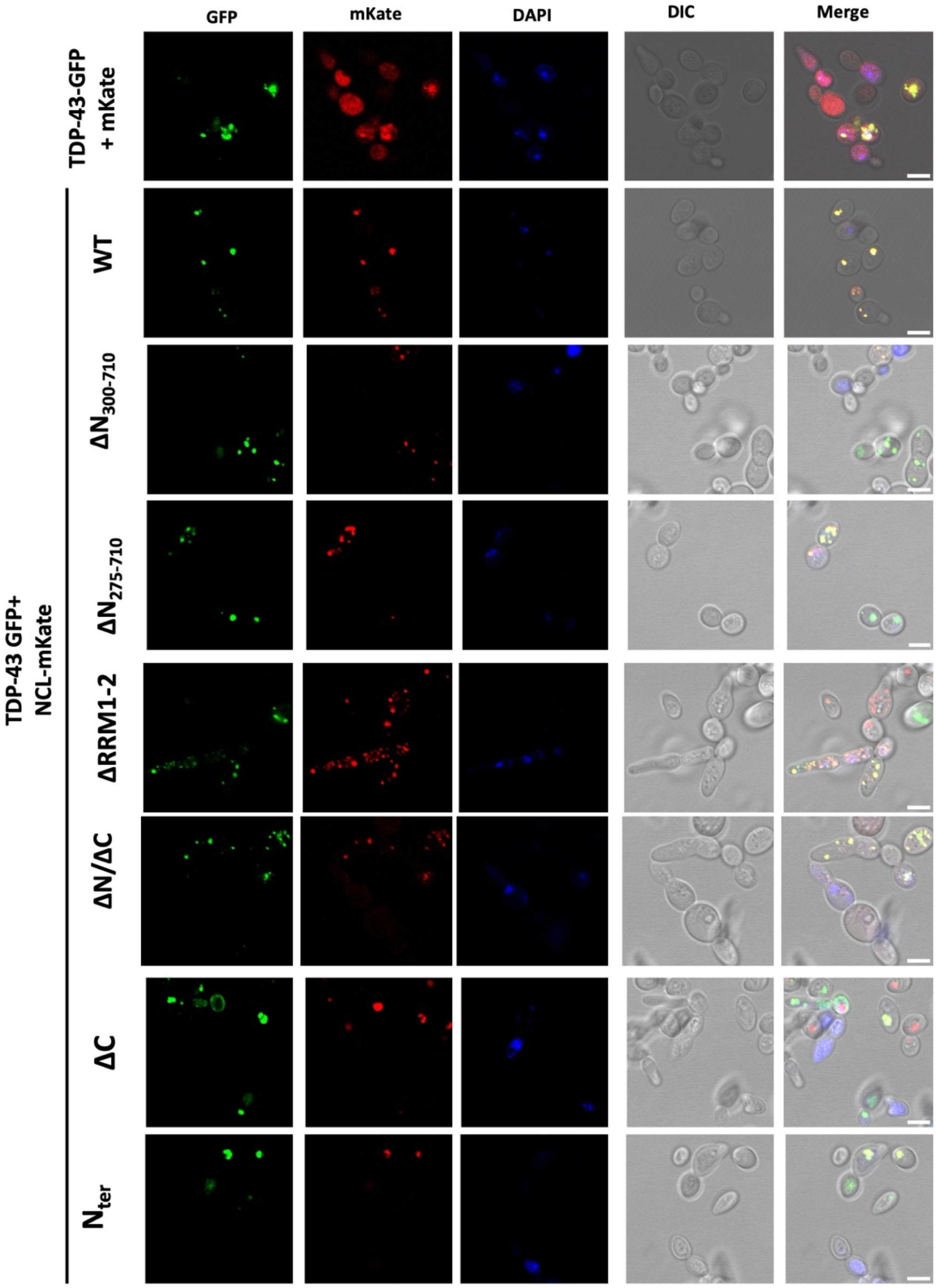

Supplementary Figure S6. Localization of TDP-43 and NCL proteins in yeast cells. CEN.PK strain was co-transformed with multi-copy plasmids expressing the TDP-43-GFP and the mKate2 red fluorescent protein alone (empty), or fused to either NCL full-length protein (WT), or the indicated mutant isoforms. After 24 hr of incubation in inducing (galactose) medium, yeast cells were fixed, and analysed by confocal microscopy to observe the distribution of either TDP-43 (GFP), or the NCL-mKate2 fusion proteins (mKate2), the nuclear staining (DAPI), the cellular morphology (DIC, *differential interference contrast*), and the merged signals (merge). Images represent yeast cells enlargements taken from fields captures from three biological replicates for each strain. Scale bar, 5 μm.

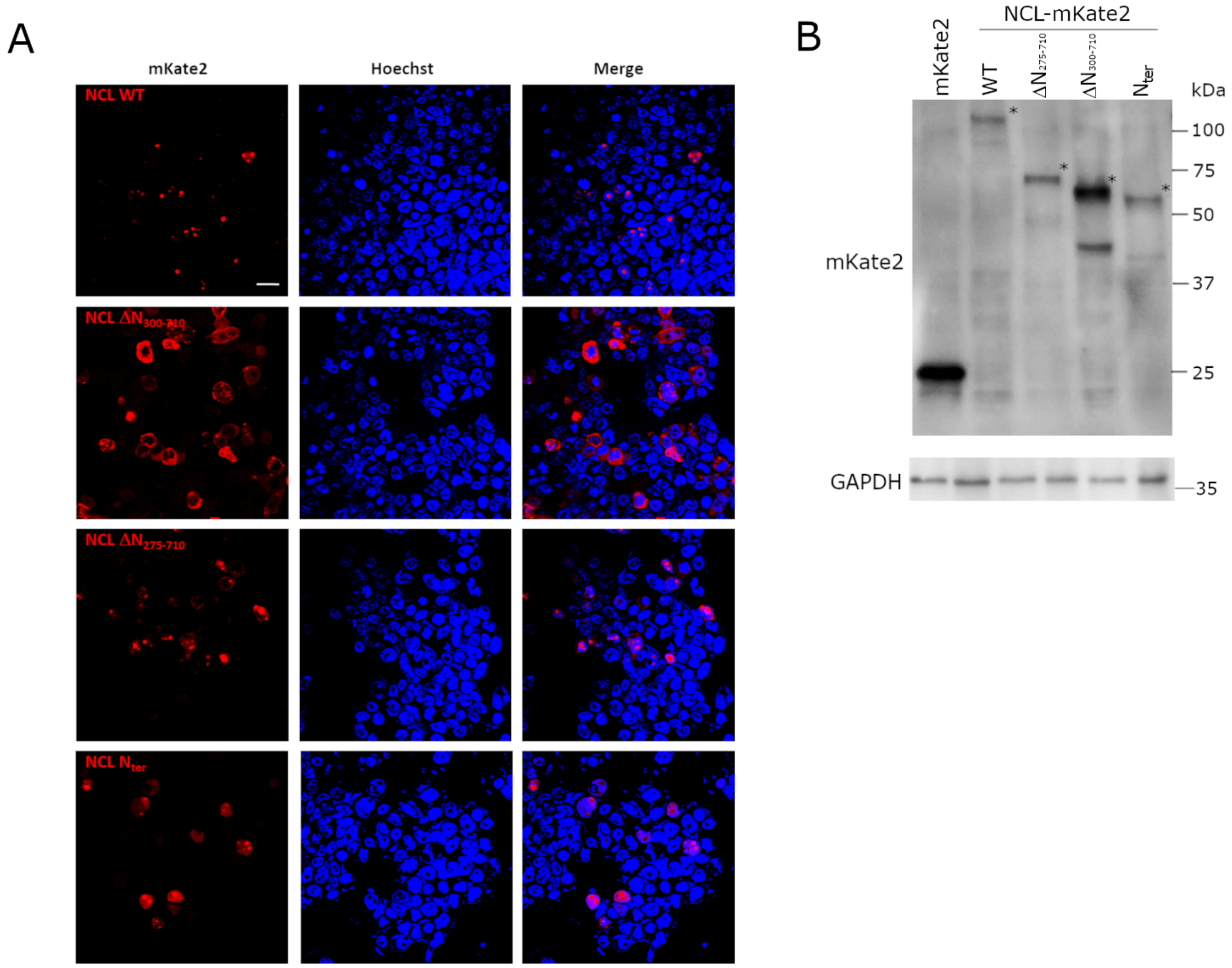

Supplementary Figure S7. Evaluation of NCL localization by confocal imaging and Western blot. A) Representative confocal images of HEK293T cells transfected with plasmids encoding for the different isoforms of NCL as indicated in the different panels. B) WB analysis of HEK293T cells transfected as in panel A using antibody against mKate2, revealing the NCL-mKate2 fusion proteins (indicated by the asterisk). As control of loading, the anti-GAPDH was used as primary antibody.

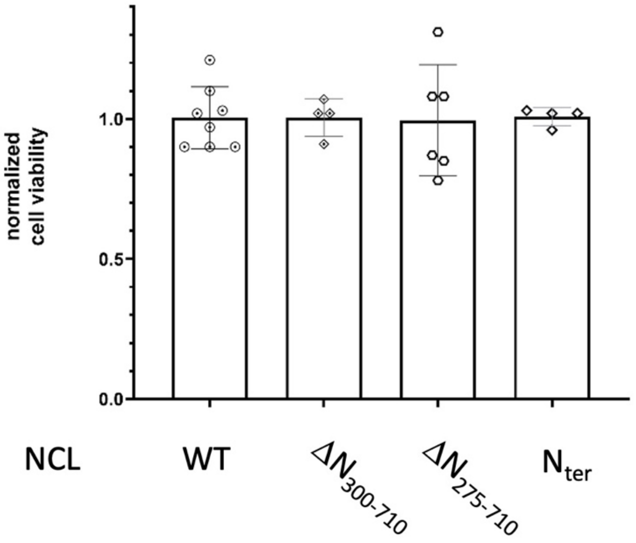

Supplementary Figure S8. The effect of NCL overexpression on cell viability was checked by MTS assay in HEK293T transiently transfected with the constructs coding for the different isoforms of NCL as indicated in Figure 5.

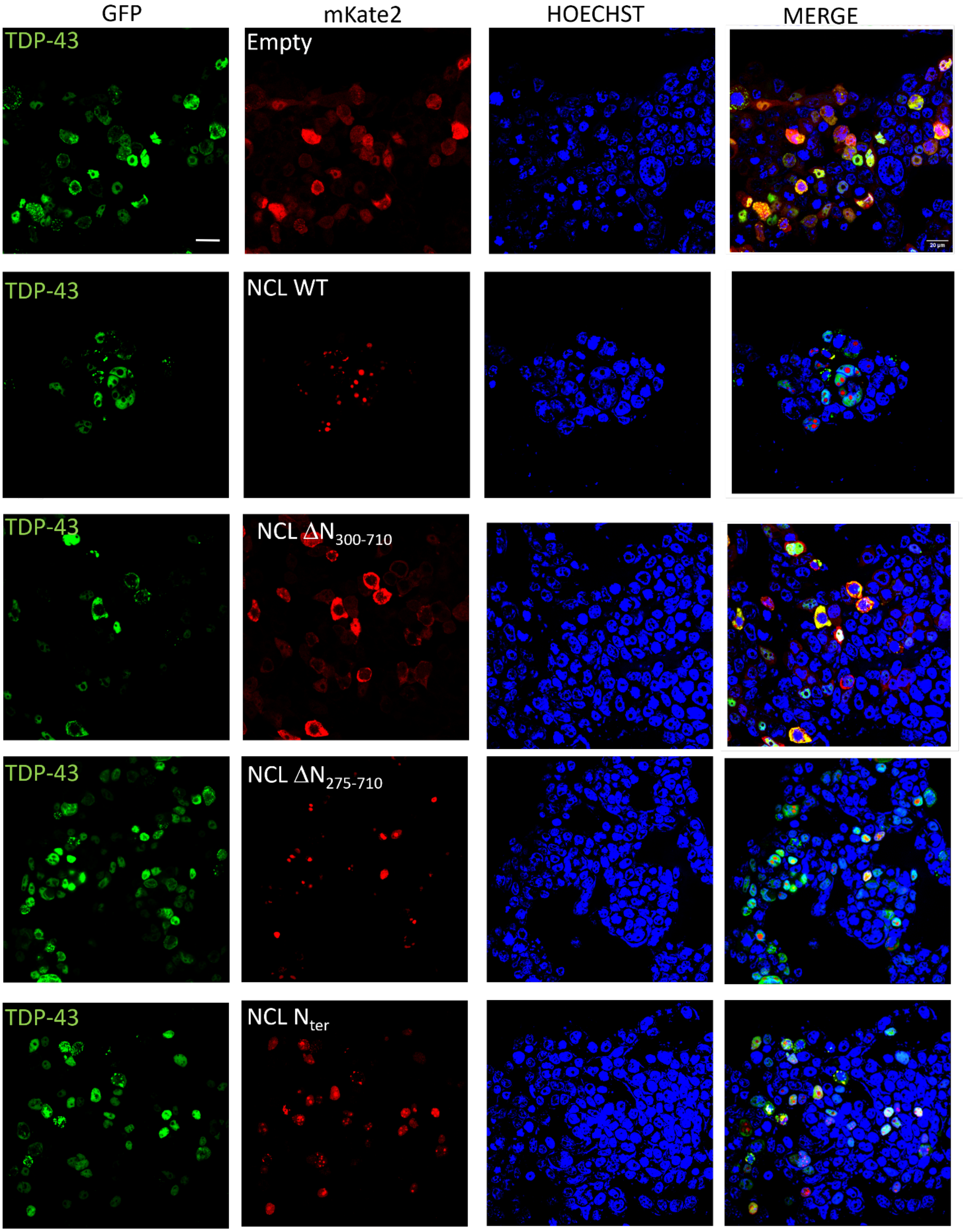

Supplementary Figure S9. Representative images of HEK293T cells co-transfected with plasmids coding for the TDP-43 G295S mutant fused to GFP and either mKate2 alone (TDP-43 + mKate2), or the different NCL-mKate2 chimeras and counterstained with the nuclear marker Hoechst. Scale 20 μm.
